# Chalcones as multitarget modulators in non-small cell lung cancer: mechanistic insights and therapeutic perspectives

**DOI:** 10.1007/s00204-026-04501-0

**Published:** 2026-07-14

**Authors:** Ellen Mayara Souza Cruz, Virginia Márcia Concato-Lopes, Mariana Barbosa Detoni, Manoela Daiele Gonçalves-Lens, Milena Cremer de Souza, Fabrício Seidy Ribeiro Inoue, Angélica Paulina Nunes, Elaine da Silva Siqueira, Ana Carolina Jacob Rodrigues, Yasmin Munhoz dos Santos-Destro, Natália Concimo Santos, João Manoel de Sousa Silva, Éder Ignácio da Silva, Maiara Voltarelli Providello, Danielle Lazarin-Bidóia, Fábio Rodrigues Ferreira Seiva, Wander Rogério Pavanelli

**Affiliations:** 1 Laboratory of Immunoparasitology of Neglected Diseases and Cancer, Department of Immunology, Parasitology and General Pathology, Center of Biological Sciences, State University of Londrina – UEL, Rodovia Celso Garcia Cid Campus, Post Box 10.011, Londrina, PR 86057-970 Brazil; 2 Laboratory of Biotransformation and Phytochemical, Department of Chemistry, Center of Exact Sciences, State University of Londrina, Londrina, PR Brazil; 3 Laboratory of Biochemistry and Experimental Morphology, Department of Chemistry and Biochemistry, São Paulo State University Júlio de Mesquita Filho, Institute of Biosciences of Botucatu, São Paulo, Brazil

**Keywords:** Epithelial–mesenchymal transition, Redox imbalance, Tumor plasticity, Therapeutic resistance, Regulated cell death, Structure–activity relationship, Selective cytotoxicity

## Abstract

Non-small cell lung cancer (NSCLC) remains the leading cause of cancer-related mortality worldwide, and the search for novel therapeutic strategies is ongoing. Chalcones, a class of naturally occurring and synthetically derived open-chain flavonoid precursors, have attracted attention because of their chemical versatility and broad biological activity. This review synthesizes current findings on the molecular mechanisms through which chalcones exert their effects in NSCLC, including modulation of oxidative stress, epithelial–mesenchymal transition, DNA damage responses, and diverse cell death pathways. Chalcones have been shown to interfere with key signaling cascades, such as TGF-β, PI3K/AKT/mTOR, JAK/STAT, and Wnt/β-catenin, with reported effects on tumor proliferation, migration, invasion, and therapy resistance. Their structural flexibility enables chemical optimization aimed at improve potency, selectivity, and pharmacokinetic profiles, supporting further preclinical investigation. Despite promising preclinical results, challenges remain regarding pharmacokinetics, bioavailability, and context-dependent off-target effects. Future research should prioritize integrated mechanistic studies, structure–activity relationship analyses, selective cytotoxicity assessment, and advanced delivery strategies to improve tumor specificity. Chalcones represent chemically versatile multitarget scaffolds for further optimization in NSCLC, particularly in strategies aimed at exploiting epithelial–mesenchymal plasticity, redox adaptation, and regulated cell death.

**Graphical Abstract:**

**Figure:**
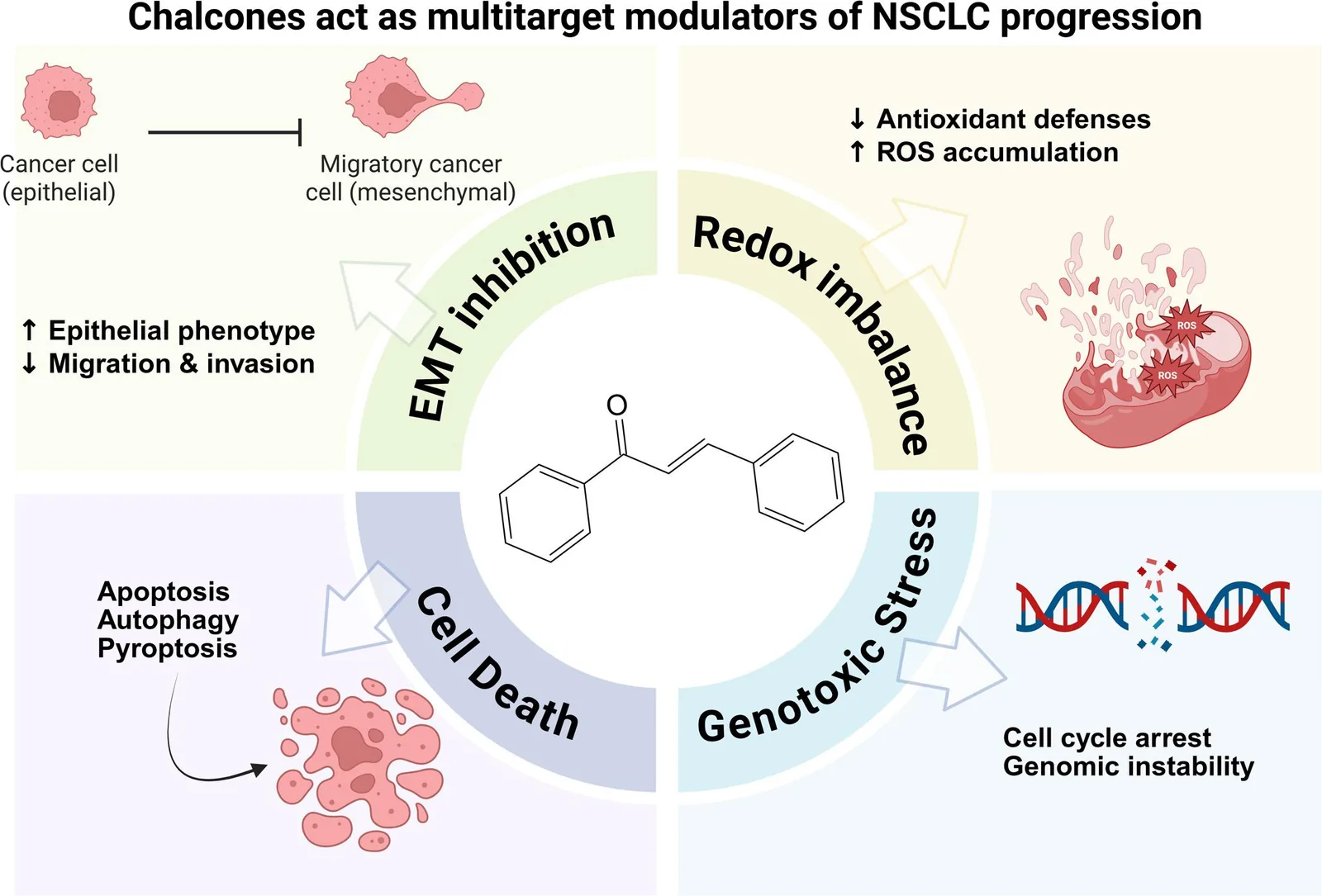

**Supplementary Information:**

The online version contains supplementary material available at https://doi.org/10.1007/s00204-026-04501-0.

## Current challenges in the lung cancer

Lung cancer is the leading cause of cancer-related death worldwide, with about 2.5 million new cases and 1.8 million deaths annually (WHO 2025). According to WHO guidelines, lung tumors are classified as small cell lung cancer (SCLC), an aggressive neuroendocrine tumor accounting for 13–15% of cases, or NSCLC, which represents about 85%. (Travis et al. 2015). NSCLC comprises three main subtypes: adenocarcinoma, squamous cell carcinoma, and large cell carcinoma. Adenocarcinoma (40%) predominates, particularly among non-smokers, originating from type II alveolar epithelial cells. Squamous cell carcinoma (25–30%) arises from bronchial squamous precursors in the central airways and is strongly linked to smoking. Large cell carcinoma (5–10%) is an undifferentiated, fast-growing form often diagnosed by exclusion and also associated with tobacco exposure (Thai et al. 2021).

Current lung cancer treatment involves multimodal strategies depending on disease stage and patient condition (Castiglioni et al. 2022). Chemotherapy remains key, especially for SCLC and advanced NSCLC. Platinum-based combinations using cisplatin or carboplatin with paclitaxel, docetaxel, vinorelbine, or gemcitabine are standard (Vasconcellos et al. 2020). However, despite improved survival, these regimens cause severe side effects and often fail due to resistance (Horiuchi et al. 2021), bringing attention to the need for new, less toxic therapies.

More recently, advances in targeted therapies and immunotherapy have provided new options for NSCLC patients, particularly those with specific genetic alterations such as EGFR mutations, ALK rearrangements, or PD-L1 overexpression. Tyrosine kinase inhibitors (TKIs) and immune checkpoint inhibitors have demonstrated improved response rates and progression-free survival compared to conventional chemotherapy in selected populations. Nevertheless, again, not all patients benefit from these approaches, and issues such as acquired resistance, high cost, and immune-related adverse events continue to limit their broad applicability, reinforcing the ongoing research for novel therapeutic agents with better efficacy and safety profiles (Liu et al. 2023a; Gálffy et al. 2024).

Despite the growing number of preclinical studies investigating the anticancer activity of chalcones in non-small cell lung cancer, the available evidence remains fragmented across distinct molecular mechanisms. To date, a comprehensive and integrative synthesis connecting chalcone-mediated modulation of epithelial–mesenchymal transition, redox homeostasis, genotoxic stress, and regulated cell death pathways has been lacking. Addressing this gap is essential to better understand the multitarget nature of chalcones and to support their rational development as therapeutic agents in NSCLC.

## Chalcones as promising compounds in NSCLC therapy

**Fig. 1:**
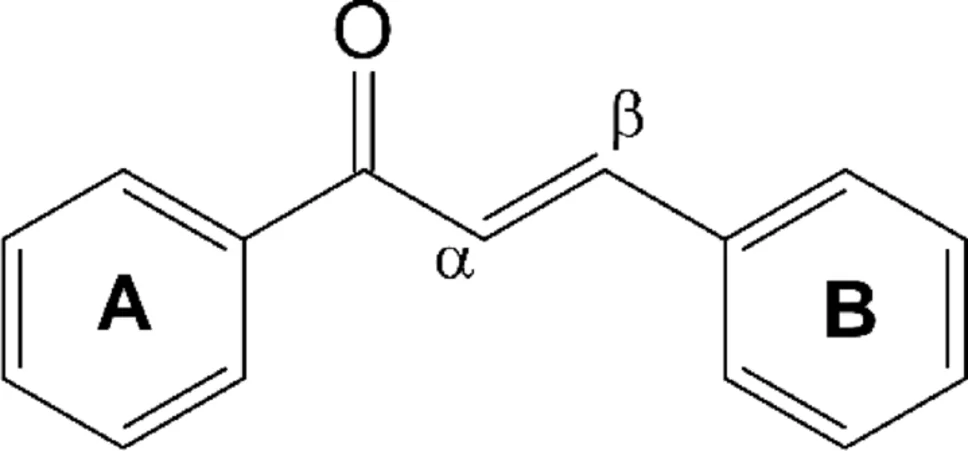
General chemical structure of chalcones. Chalcones are characterized by a 1,3-diphenyl-2-propen-1-one scaffold composed of two aromatic rings (A and B) connected by an α,β-unsaturated carbonyl system. This central enone moiety is responsible for the electrophilic properties of chalcones and serves as a key determinant of their biological activity. Structural modifications on rings A and B can modulate physicochemical properties and influence their interaction with multiple molecular targets

Natural products have emerged as valuable sources of anticancer agents, and chalcones are among the most promising. Chalcones (1,3-diphenyl-2-propen-1-one) (Fig. 1), are flavonoid precursors with anti-inflammatory, antioxidant, antimicrobial, and anticancer properties. Their α,β-unsaturated carbonyl system contributes to strong chemical and biological activity (Bukhari et al. 2012; Zhuang et al. 2017). They are biosynthesized from L-phenylalanine via p-coumaroyl-CoA, which reacts with malonyl-CoA under chalcone synthase catalysis to form the chalcone backbone (Dao et al. 2011). Chalcones can be converted into aurones or naringenin, a key flavonoid precursor, and play roles in signaling and symbiosis (Jiang et al. 2015).

Recent preclinical studies show that natural and synthetic chalcones exert strong anticancer effects in NSCLC models by inducing apoptosis, arresting the cell cycle, and inhibiting angiogenesis and tumor progression (Gao et al. 2020; Ouyang et al. 2021; WalyEldeen et al. 2023) (Suppl. Table 1). These activities point to chalcones as potential modulators of key pathways involved in tumor growth and resistance. Moreover, chalcones offer unique opportunities for chemical modification, allowing the design of derivatives with enhanced selectivity, potency, and pharmacokinetic properties.

This review summarizes current knowledge on chalcones in NSCLC, focusing on mechanisms of action, structural modifications enhancing their activity, and promising synthetic derivatives. It also outlines existing limitations and future directions for developing chalcone-based therapies.

### Structural features associated with chalcone activity in NSCLC: qualitative structure–activity trends

**Fig. 2:**
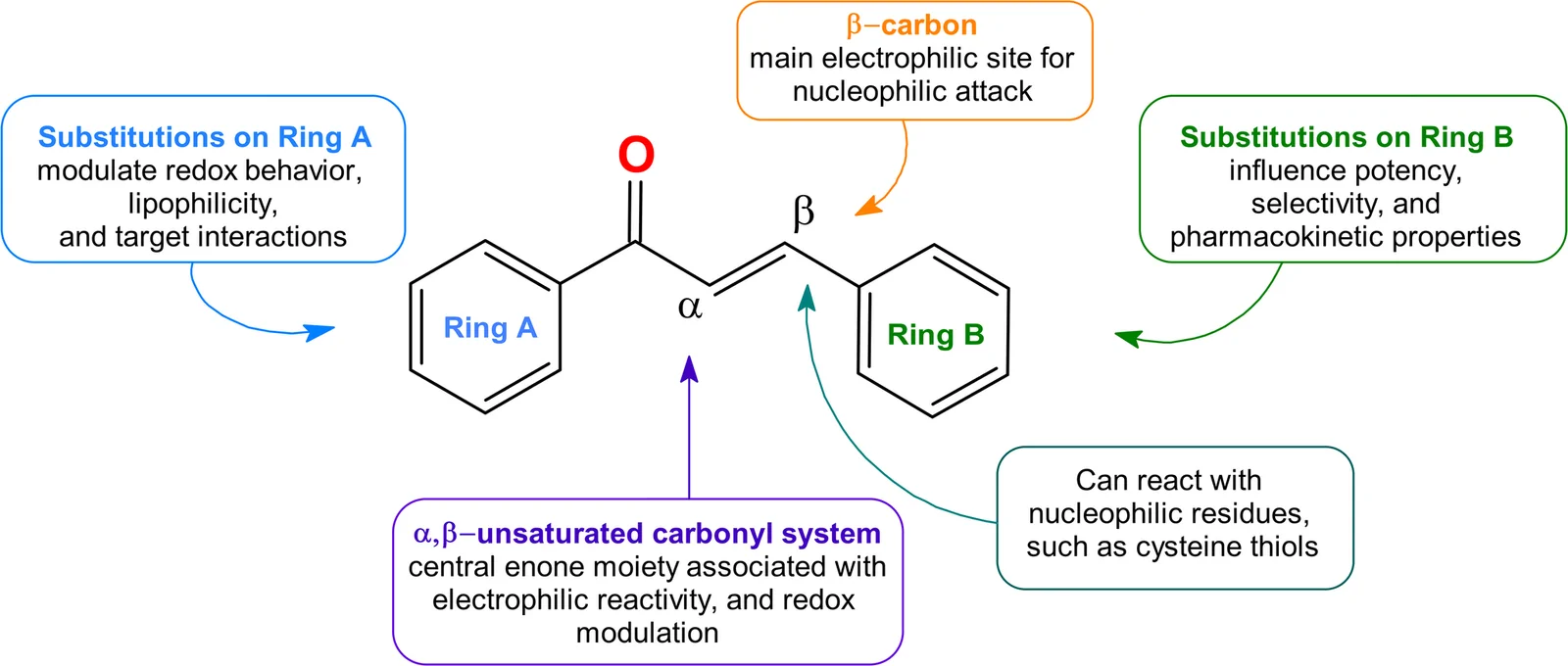
Structural determinants associated with chalcone activity in NSCLC. Schematic representation of the main structural features that influence the biological activity of chalcones. The α,β-unsaturated carbonyl system constitutes the central electrophilic moiety and the β-carbon acts as the primary site for nucleophilic attack. Substitutions on ring A may modulate redox behavior, lipophilicity, and target interactions, whereas modifications on ring B can influence potency, selectivity, and pharmacokinetic properties. The electrophilic enone system may also interact with nucleophilic residues, particularly cysteine thiols, contributing to redox modulation and multitarget biological effects

The anticancer activity of chalcones in NSCLC is strongly influenced by the structural tunability of the 1,3-diphenyl-2-propen-1-one scaffold. This scaffold allows modifications in both aromatic rings and in the α,β-unsaturated carbonyl system, thereby modulating redox behavior, target interactions, and selectivity. (Bukhari et al. 2012; Zhuang et al. 2017). Although the heterogeneity of experimental models precludes a definitive quantitative SAR (QSAR), current evidence supports a qualitative framework based on three interconnected structural determinants: electrophilic reactivity of the enone system, substitution patterns on the aryl rings, and structural hybridization (Fig. 2; Suppl. Table 2).

In this context, the α,β-unsaturated carbonyl system acts as a Michael acceptor, meaning that it behaves as an electron-deficient electrophilic site capable of reacting with nucleophilic groups, especially cysteine thiols, through conjugate addition (Bukhari et al. 2012; Zhuang et al. 2017).While this property explains the multitarget interference of chalcones, it represents a critical toxicological threshold. Excessive or poorly controlled electrophilicity may lead to nonspecific protein modification, thiol depletion, and off-target cytotoxicity (Kachadourian and Day 2006; Kachadourian et al. 2007a, b; Bukhari et al. 2012; Zhuang et al. 2017). In NSCLC models like A549 cells, derivatives such as 2′-hydroxychalcone induce significant glutathione (GSH) depletion, sensitizing tumor cells to oxidative stress. This mechanism exploits the redox vulnerability strategy: since NSCLC cells often operate under elevated basal oxidative stress, the targeted electrophilic attack of chalcones can push them beyond their antioxidant capacity, whereas normal cells, with higher GSH reserves, may maintain homeostasis. Thus, the therapeutic window of chalcones therefore depends on balancing electrophilic potency sufficient to induce tumor-selective stress while avoiding indiscriminate systemic toxicity (Kachadourian and Day 2006; Kachadourian et al. 2007a, b).

Substituents on the aryl rings fine-tune the electronic distribution and physicochemical properties of the chalcone scaffold (Bukhari et al. 2012; Zhuang et al. 2017; Ouyang et al. 2021; WalyEldeen et al. 2023). Since the nomenclature of rings A and B may vary among studies, we refer here to the aryl ring adjacent to the carbonyl group and the β-aryl ring of the enone system.

Hydroxylated chalcones, such as butein (2′,3,4,4′-tetrahydroxychalcone), are consistently associated with enhanced redox cycling. These polyhydroxylated derivatives engage in hydrogen-bonding networks and trigger ROS-mediated endoplasmic reticulum (ER) stress via PERK/eIF2α/CHOP signaling pathway, leading to G2/M arrest and apoptosis (Di et al. 2019; Sulaiman et al. 2021).

Conversely, methoxylated chalcones, such as 2′-hydroxy-2,3,4′,6′-tetramethoxychalcone, Flavokawain B (2′-hydroxy-4′,6′-dimethoxychalcone), and chalcones linked to the pyrazolo[1,5-a]pyrimidines, typically exhibit increased lipophilicity and improved cellular uptake. Methoxy groups appear to favor interactions within protein-binding pockets, cause mTOR inhibition, induce autophagy, oncogenic signaling disruption, and cell cycle arrest (Rao et al. 2010; Bagul et al. 2017; Hseu et al. 2019). The coexistence of hydroxyl and methoxy groups (e.g., 2′-hydroxy-4′,5′-dimethoxychalcone) can be particularly potent, as seen in the activation of the death receptor 5 (DR5) pathway via the PERK–ATF4–CHOP axis. This profile suggests that the spatial arrangement of these groups shapes intramolecular hydrogen bonding and target engagement. Similarly, Flavokawain B induced ROS-mediated autophagic and apoptotic cell death in A549 and H1299 cells through mTOR inhibition and ATG4B-related mechanisms (Yang et al. 2013; Hseu et al. 2019).

Additional substituents further illustrate the context-dependency of chalcone SAR. Halogenation, particularly with chlorine or bromine, can modulate membrane permeability, electronic distribution, and binding orientation. The brominated hybrid ZW97((E)-1-(4-(3-((1,3,4-thiadiazol-2-yl)thio)propoxy)phenyl)-3-(4-bromophenyl)prop-2-en-1-one) reversed EMT-associated phenotypes by inhibiting c-Met signaling (Zhao et al. 2022). Compound 8 ((E)-N-(4-(3-(2-chlorophenyl)acryloyl)phenyl)acrylamide), which contains a chlorinated aromatic group and an electrophilic chalcone-like structure, induces ROS accumulation and caspase-3-mediated pyroptosis in NCI-H460 cells (Zhu et al. 2018). Nitro groups, as seen in Pyrazolyl chalcone 7b ((E)-1-(3-(furan-2-yl)-5-methyl-1-(4-nitrophenyl)-1H-pyrazol-4-yl)-3-(thiophen-2-yl)prop-2-en-1-one), strongly influence electronic properties and were associated with G0/G1 arrest (Mohamed et al. 2023). However, nitroaromatic substituents also represent a structural alert for potential genotoxicity, as their metabolic activation may lead to DNA-reactive intermediates (Eichenbaum et al. 2009; Mohamed et al. 2023). While not confirming toxicity for all nitrochalcone derivatives, it supports the need for compound-specific genotoxicity assessments.

Hybridization strategies combine the chalcone core with heterocyclic, metal-based, or bioactive moieties to expand molecular interactions. These hybrids (e.g., PCH-1 (1-[3-(6-methoxy-2H-1,3-benzodioxol-5-yl)-5-(4-methoxyphenyl)-4,5-dihydro-1H-pyrazol-1-yl]ethan-1-one), HYQ97 Tert-butyl-4-((2-(1-(4-cinnamoylphenyl)-1H-1,2,3-triazol-4-yl)ethanethioyl)thio)piperazine-1-carboxylate), and complex 17a (OC-6-33)-diamminetrichlorido((E)-3-(4-(3-oxo-3-(3,4,5-trimethoxyphenyl)prop-1-en-1-yl)-1H-indol-1-yl)propanoate)platinum(IV)) do not replace the intrinsic reactivity of the chalcone core but complement it by adding molecular recognition elements that improve protein-target interaction and cellular distribution (Maciejewska et al. 2022; Liu et al. 2023b).

The most relevant SAR question is how structural modifications drive selectivity for NSCLC cells over normal bronchial epithelial cells. The dual behavior of chalcones, i.e. activating the cytoprotective Nrf2/HO-1 axis in normal cells while inducing ROS-dependent apoptosis in A549 cells, suggests a dose- and structure-dependent exploitation of tumor redox vulnerability (Kumar et al. 2011; Zhao et al. 2017). From a toxicological perspective, optimization must prioritize controlled electrophilicity to minimize damage to metabolically active organs, like the liver. The most promising chalcone derivatives are those that integrate moderate reactivity with substitution patterns that fine-tune the crosstalk between oxidative stress, DNA damage, and EMT reversal. This balance defining a narrow but exploitable therapeutic window in NSCLC (Kachadourian and Day 2006; Kachadourian et al. 2007a, b).

## Modulation of the epithelial–mesenchymal transition by chalcones

The epithelial–mesenchymal transition (EMT) is a biological process in which epithelial cells acquire mesenchymal traits, enhancing motility and invasiveness. While essential for development and tissue repair, EMT activation is a hallmark of cancer progression, driving metastasis (Roche 2018; Hao et al. 2019; Hwang et al. 2022; Youssef et al. 2024). Several studies have demonstrated that chalcone-derived compounds can interfere with EMT by targeting key signaling pathways.

Chalcone-derived compounds have been shown to modulate EMT by regulating critical signaling cascades, including TGF-β, Wnt/β-catenin, and PI3K/AKT pathways, which are frequently dysregulated in NSCLC. By downregulating mesenchymal markers such as N-cadherin, vimentin, and Snail while upregulating epithelial markers like E-cadherin, these compounds can inhibit cell migration, invasion, and metastatic potential. Moreover, chalcone derivatives have been reported to sensitize resistant NSCLC cells to chemotherapy by reversing EMT-associated drug resistance, highlighting their dual role in controlling both metastasis and treatment refractoriness.

### TGF-β and related kinases

Transforming growth factor β1 (TGF-β1), abundant in the metastatic tumor microenvironment (TME), is a central inducer of EMT (Hao et al. 2019; Hwang et al. 2022). Chalcone derivatives have shown potent inhibition of this pathway (Jeong et al. 2019; Shi et al. 2024). Screening of 84 analogues identified compound 67 as a TGF-β1 inhibitor that reduced migration and invasion in A549 cells undergoing TGF-β1-induced EMT (Jeong et al. 2019). CTI-82, a related derivative, produced similar effects in A549 and HaCaT cells by blocking Smad2 phosphorylation, restoring E-cadherin, and reducing N-cadherin, vimentin, PAI-1, MMP-2, MMP-9, and phospho-ERK1/2 (Jeong et al. 2020). Likewise, PCH-1 suppressed TGF-β1-induced EMT in A549 cells by downregulating N-cadherin and vimentin (Maciejewska et al. 2022).

Beyond TGF-β, chalcones also act on other kinases controlling EMT. The compound (E)-1-(4-(3-((1,3,4-thiadiazol-2-yl)thio) propoxy)phenyl)-3-(4-bromophenyl) prop-2-en-1-one, a chalcone–1,3,4-thiadiazole hybrid, reversed EMT in NCI-H460 cells via c-Met kinase inhibition(Zhao et al. 2022). Isoliquiritigenin and its metabolite 2,4,2′,4′-tetrahydroxychalcone suppressed cytoskeletal remodeling and focal adhesion through FAK and Src inhibition, reducing lung tumor growth and metastasis in vivo (Chen et al. 2018). Licochalcone A from *Glycyrrhiza inflata* inhibited migration and invasion of A549 and NCI-H460 cells by suppressing Sp1 and the AKT pathway, leading to downregulation of MMP-1 and MMP-3 (Huang et al. 2014).Consistently, knockdown of the SP1/Syncytin-1 axis in A549 cells suppresses AKT and ERK1/2 phosphorylation, reverses EMT, and reduces migration and invasion, underscoring the central role of Sp1–AKT signaling in NSCLC progression (Li et al. 2019).

These findings demonstrate the multifaceted role of chalcone derivatives in modulating kinase-driven EMT. By simultaneously acting on multiple signaling nodes, such as Smad-dependent TGF-β pathways, receptor tyrosine kinases like c-Met, and downstream effectors including FAK, Src, and AKT, chalcones interfere with the mechanisms that promote NSCLC cell migration, invasion, and metastasis. Their ability to restore epithelial marker expression while reducing mesenchymal traits indicates potential not only as anti-metastatic agents but also as adjuvants to increase the effectiveness of conventional therapies. Overall, the structural versatility of chalcones allows fine-tuning of their kinase inhibitory effects, offering a promising platform for the development of targeted therapies against EMT in lung cancer.

### Other kinase and pathway targets

Maternal embryonic leucine zipper kinase (MELK), implicated in EMT and metastasis (McDonald and Graves 2020; Tan et al. 2023; Wang et al. 2024), was recently identified as a target of the chalcone–tetramethylpyrazine hybrid HCTMPPK (E)-1-(E)-1-(2-hydroxy-5-chlorophenyl)-3-(3,5,6-trimethylpyrazin-2-yl)-2-propen-1. This compound markedly reduced A549 cell migration, invasion, and MELK expression, while inducing apoptosis via Bax and caspase-3 upregulation. In vivo, this compound suppressed tumor growth without systemic toxicity (Ma et al. 2024).

Chalcones also inhibit the JAK2/STAT3 pathway, often overactivated during EMT (Jin 2020). Metochalcone, 2′,4,4′-trimethoxychalcone, reduced migration and invasion in BT549 and A549 cells by blocking this pathway (Zhou et al. 2024), while 3,4,2′,4′-tetrahydroxychalcone (butein) suppressed the c-Src/JAK2/STAT3 cascade in NSCLC (Di et al. 2019; Sulaiman et al. 2021). Polyoxygenated chalcones antagonizing the farnesoid X receptor (FXR) also disrupted the FXR/IL-6/JAK2/STAT3 axis; compound 2-methoxy-2′-hydroxy-4′-(4″-methoxy-4″-oxo-E-crotonyl) chalcone (6p) significantly inhibited migration and invasion in H460, H1975, and H1299 cells (Niu et al. 2022). FXR has recently been shown to drive NSCLC metastasis through an FXR/IL-6/IL-6ST/JAK2/STAT3 axis, further supporting FXR as a relevant target for FXR-antagonistic chalcones such as 6p (Jin et al. 2024).

The PI3K/Akt/mTOR signaling pathway is another key target, given its central role in driving EMT through the activation of transcription factors such as Snail, Slug, and Twist (Moghbeli 2024; Wang et al. 2024). Cardamonin inhibited AKT/mTOR phosphorylation, reducing N-cadherin and ZEB1 while restoring E-cadherin in lung cancer cells (Zhou et al. 2019). Similarly, hydroxysafflor yellow A reversed LPS-induced EMT by regulating PI3K/AKT/mTOR and ERK/MAPK signaling and suppressing inflammatory mediators (Jiang et al. 2019).

Overall, these studies demonstrate that chalcone-derived compounds can act on multiple kinases and signaling pathways that regulate EMT, metastasis, and tumor progression. By modulating MELK, JAK2/STAT3, PI3K/AKT/mTOR, and related cascades, chalcones not only inhibit cell migration and invasion but also restore epithelial characteristics and promote apoptosis. This multitarget activity reinforces the potential of chalcones as effective and less toxic therapeutic agents for NSCLC, addressing critical mechanisms of metastasis and therapy resistance.

### Transcription factors and other regulators

Chalcones also modulates EMT-related transcription factors. Panduratin A (PA) inhibited NF-κB translocation, decreasing MMP-2 secretion and invasion in A549 cells (Cheah et al. 2011). The synthetic 4′-hydroxyl-3,4-dimethoxyl-3′-methoxylchalcone (chalcone 9X) reduced migration, invasion, and EMT marker expression through FOXM1 inhibition in both hepatic and lung cancer cells (Dong et al. 2018; Li et al. 2022). Pyrazolyl chalcone 1-[5-methyl-1-(4-nitrophenyl)-3-(thiophen-2-yl)-1H-pyrazol-4-yl]-3-phenylprop-2-en-1-one (7 g) downregulated FOXG1, another EMT regulator linked to the Wnt/β-catenin pathway (Zheng et al. 2021; Helmy et al. 2022).

The Wnt/β-catenin signaling pathway is a central regulator of epithelial–mesenchymal transition (EMT) (Liu et al. 2022a). In this context, two benzofuran-substituted chalcone derivatives, designated as compound 1 and compound 2, were evaluated in combination with niclosamide and demonstrated a marked inhibitory effect on cell migration and invasion. These compounds significantly downregulated EMT-associated genes, including VIM, CDH2, and SNAI1, while restoring E-cadherin (CDH1) expression, indicating effective suppression of EMT through modulation of Wnt/β-catenin signaling (Erturk et al. 2023). PCH-1 also inhibited β-catenin and human telomerase reverse transcriptase (hTERT), affecting EMT and cancer stemness (Maciejewska et al. 2022).

Inhibition of Hsp90, a chaperone stabilizing PI3K/AKT, RAS/RAF/ERK, and Wnt/β-catenin signaling, further demonstrates chalcones’ pleiotropy. The triazole–dithiocarbamate hybrid HYQ97 suppressed migration and invasion in A549 cells by downregulating N-cadherin and vimentin while increasing E-cadherin (Hu et al. 2021).

**Fig. 3:**
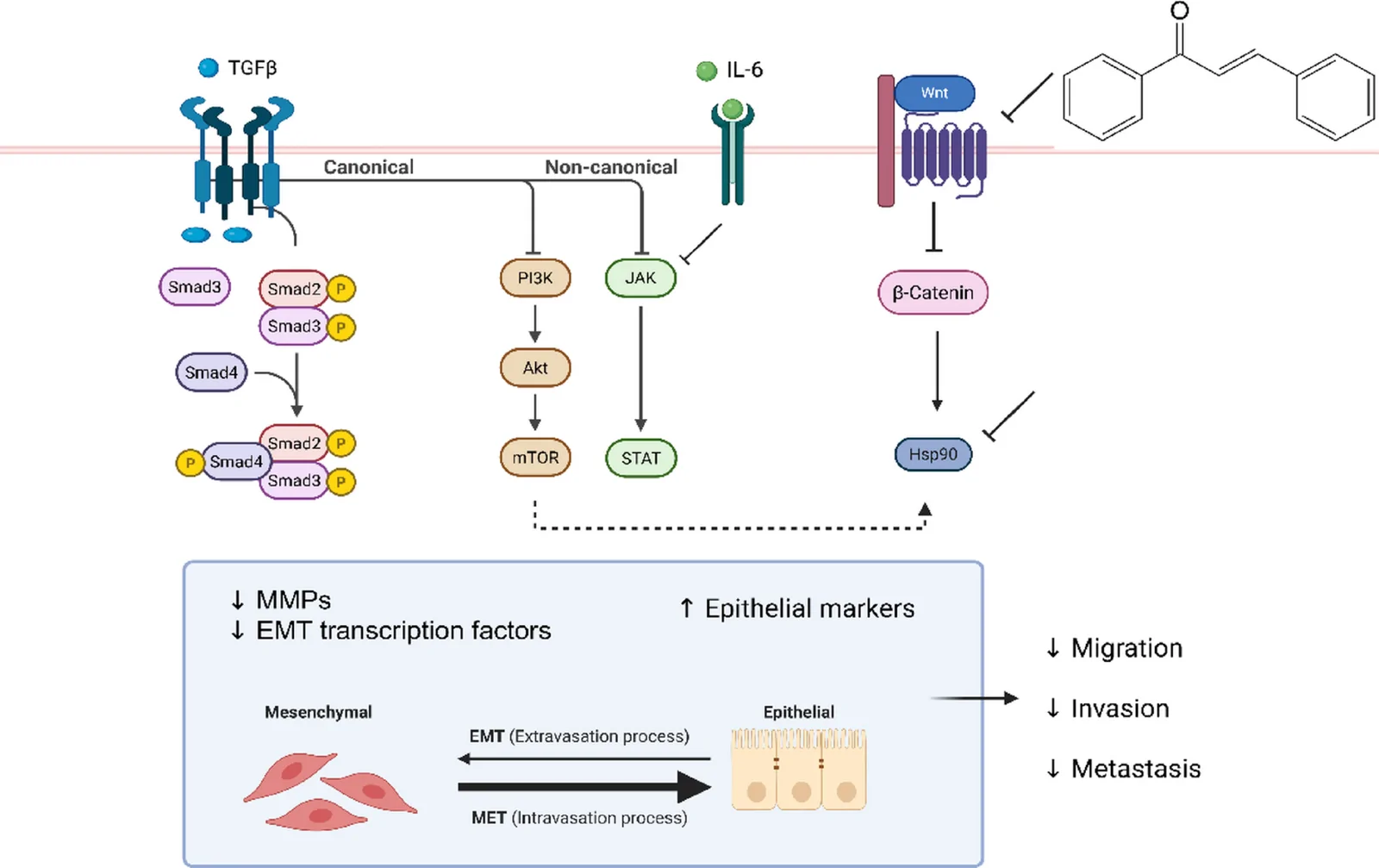
Chalcone-mediated inhibition of epithelial–mesenchymal transition in non-small cell lung cancer. Chalcones interfere with the epithelial-mesenchymal transition (EMT) process by modulating key signaling pathways involved in tumor progression, including TGF-β/Smad, PI3K/Akt/mTOR, JAK/STAT and Wnt/β-catenin signaling. By destabilizing EMT-associated signaling networks and molecular chaperones such as Hsp90, chalcones suppress mesenchymal traits, restore epithelial characteristics and reduce migration, invasion and metastatic potential in non-small cell lung cancer cells

Together, these findings indicate that chalcones interfere with epithelial–mesenchymal transition in non-small cell lung cancer by acting on multiple transcription factors, kinase-driven signaling pathways, and molecular chaperones. By simultaneously modulating NF-κB, FOXM1, FOXG1, Wnt/β-catenin, PI3K/AKT, JAK2/STAT3, and Hsp90, chalcone derivatives not only inhibit migration and invasion but also restore epithelial characteristics, reduce cancer stemness, and attenuate EMT-associated therapeutic resistance. This broad spectrum of activity underscores their potential as lead compounds for the development of therapies targeting EMT-driven metastasis in NSCLC (Fig. 3).

## Chalcones as inducers of redox imbalance

Oxidative stress arises from an imbalance between reactive oxygen species (ROS) production and antioxidant defenses, disrupting redox signaling and causing molecular damage (Pham-Huy et al. 2008; Lobo et al. 2010; Sies 2015; Mittler 2017). Mitochondria, peroxisomes, and the ER are the main ROS sources. At moderate levels, ROS act as signaling molecules (Sauer and Wartenberg 2005; Finkel 2011), but in tumors, they accumulate due to increased metabolism, oncogenic signaling, and hypoxia (Cheung and Vousden 2022; An et al. 2024). Exceeding the oxidative threshold can overwhelm antioxidant systems, leading to apoptosis or necrosis (Jomova and Valko 2011; Fuchs-Tarlovsky 2013).

Chalcones, both natural and synthetic, can elevate intracellular ROS, inducing redox-dependent cell death (Kim et al. 2010; Coskun et al. 2017; Gil et al. 2019; Maciejewska et al. 2022; Xiao et al. 2023). Among natural compounds, isobavachalcone triggers mitochondrial calcium overload, membrane depolarization, and necrotic death in breast and lung cancer cells, also suppressing tumor growth in vivo (Xiao et al. 2023). Synthetic derivatives such as PCH-1 (Maciejewska et al. 2022) and 3-[4-(dimethylamino)phenyl]-1-(7-ethoxy-1-benzofuran-2-yl)prop-2-en-1-one (3a)(Coskun et al. 2017) cause ROS-dependent mitochondrial dysfunction and apoptosis across several cancer types. Other derivatives, including 2-hydroxy-3′,5,5′-trimethoxychalcone (DK-139) (Gil et al. 2019) and 4-(p-toluenesulfonylamino)-3,4-dihydroxychalcone (TSHDC) (Kim et al. 2010), also promote ROS accumulation. Notably, TSHDC induces oxidative stress through c-Myc activation, as silencing of c-Myc reverses this effect.

Beyond ROS induction, chalcones regulate oxidative stress–related processes like autophagy (Wang et al. 2014; Zhou et al. 2014; Hseu et al. 2019). 2′-Hydroxy-4′,6′-dimethoxychalcone (Flavokawain B) induced autophagy in A549 and H1299 cells by promoting LC3-II accumulation and mTOR inhibition while linking this effect to ROS-mediated ATG4B regulation. ROS scavenging by N-acetylcysteine reversed cytotoxicity, confirming the central role of oxidative stress (Hseu et al. 2019). In a complementary approach, (Zhu et al. 2018) described a synthetic chalcone analogue, compound 8 ((E)-N-(4-(3-(2-chlorophenyl)acryloyl)phenyl) acrylamide) that elevates ROS and triggers caspase-3-mediated pyroptosis in NCI-H460 cells (Zhu et al. 2018).

ROS also contribute to ER stress (Disbrow et al. 2005; Schleicher et al. 2010; Cao et al. 2022). The platinum(IV)–chalcone complex 17a, (OC-6-33)-diamminetrichlorido((E)-3-(4-(3-oxo-3-(3,4,5-trimethoxyphenyl)prop-1-en-1-yl)-1H-indol-1-yl)propanoate)platinum(IV), induced ROS accumulation, mitochondrial depolarization, and ER stress in cisplatin-resistant A549/CDDP cells (Liu et al. 2022b, 2023b). Butein also increased ROS levels, altered antioxidant enzyme activity, and activated the PERK/eIF2α/CHOP branch of the unfolded protein response (Di et al. 2019; Zhang et al. 2019). Inhibition of ROS generation or ER stress partially rescued cell viability, confirming the interdependence of these pathways. 2′-Hydroxy-4′,5′-dimethoxychalcone was shown to induce ROS-dependent ER stress through activation of the PERK/p-eIF2α/ATF4/CHOP signaling axis, leading to death receptor 5 (DR5) upregulation and apoptosis in multiple non-small cell lung cancer cell lines, including H157, H460, H1792, H322M, and H358. Similarly, Licochalcone A triggers ER stress via the PERK/eIF2α/ATF4/CHOP pathway in A549 and H1299 cells, promoting DR5 expression and apoptotic cell death (Yang et al. 2013; Tang et al. 2016; Qiu et al. 2017a; Fan et al. 2023).

Collectively, these findings indicate that chalcones exert multifaceted cytotoxic effects through redox modulation, integrating mitochondrial dysfunction, ER stress, and autophagy. By simultaneously increasing ROS, impairing antioxidant defenses, and activating stress-response pathways, chalcones shift cancer cells beyond their oxidative threshold, triggering apoptosis, necrosis, or autophagy-dependent cell death. This dual capacity to enhance ROS generation while modulating downstream signaling underscores chalcones’ potential as selective anticancer agents, particularly for tumors with elevated basal oxidative stress or resistance to conventional therapies.

### Antioxidant defense

Redox homeostasis depends on enzymatic (SOD, CAT, peroxidases, peroxiredoxins) and non-enzymatic (GSH, vitamins C and E) antioxidants (Basak et al. 2017). Heme oxygenase-1 (HO-1) also contributes through heme degradation and ROS production (Consoli et al. 2021). While many chalcones act as pro-oxidants, others exhibit antioxidant properties. For instance, pyrazoline derivative 1-(1′-(4-Bromophenyl)-3′-(4-methoxyphenyl)-5-(thiophen-2-yl)-3,4-dihydro-1′H,2H-[3,4′-bipyrazol]-2-yl)ethan-1-one (9d) showed both cytotoxicity in A549 cells and dose-dependent DPPH radical scavenging (Rana et al. 2023).

The transcription factor Nrf2 orchestrates the antioxidant response by upregulating HO-1, NQO1, and GCLM (Basak et al. 2017). Chalcone (E)-1-(2-methoxyphenyl)-3-(2-(trifluoromethyl)phenyl)prop-2-en-1-one (2b) enhanced Nrf2-regulated genes in bronchial BEAS-2B cells and in vivo (Kumar et al. 2011). Likewise, compound 1, 1'-(3-methyl-1H-indole-1, 2-diyl) bis (3-phenylprop-2-en-1-one) (3d) increased ROS generation while activating the Nrf2/HO-1 axis and inducing apoptosis in A549 cells (Zhao et al. 2017). Chalcones also modulate glutathione (GSH), a key redox buffer and substrate for glutathione peroxidase (Liu et al. 2022b). Hydroxychalcone (2′-HC) and related derivatives depleted up to 90% of intracellular GSH in A549 cells (Kachadourian and Day 2006), sensitizing them to oxidative stress. Subsequent studies showed that 2′-HC, 2′,5′-DHC, and 4′-HC enhanced cisplatin cytotoxicity by further depleting GSH and intensifying mitochondrial ROS production (Kachadourian et al. 2007a, b).

Moreover, chalcones can differentially regulate the balance between protective and cytotoxic responses depending on cellular context. While activation of the Nrf2/HO-1 pathway may shield normal cells from oxidative damage, sustained or excessive stimulation in cancer cells often triggers apoptosis or ferroptosis through ROS overproduction. Certain chalcone derivatives simultaneously suppress enzymatic antioxidants like SOD and CAT while disrupting thiol redox buffers, creating a selective vulnerability in tumor cells with high basal oxidative stress. This dual modulation, enhancing pro-oxidant effects while partially engaging antioxidant defense, allows chalcones to finely tune redox signaling, tipping cancer cells toward cell death without broadly harming normal tissues (Kumar et al. 2011).

**Fig. 4:**
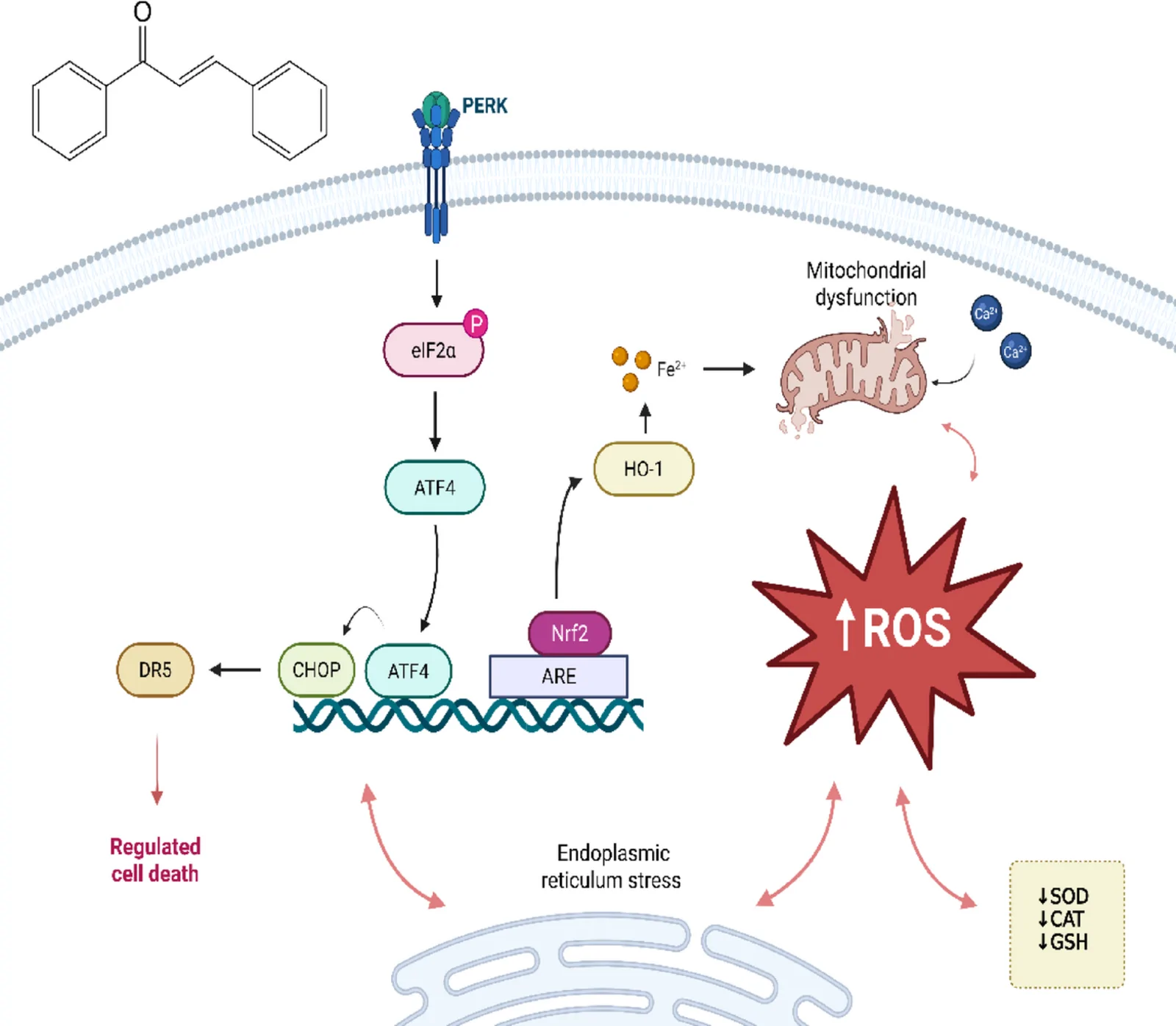
Chalcone-induced redox imbalance and stress signaling in non-small cell lung cancer. Chalcones disrupt redox homeostasis in non-small cell lung cancer cells by promoting the accumulation of reactive oxygen species and impairing the antioxidant defense system. This imbalance in redox triggers endoplasmic reticulum stress signaling, primarily through the PERK–ATF4/CHOP axis, inducing mitochondrial dysfunction and amplifying oxidative stress. The convergence of these stress responses drives tumor cells beyond the threshold for oxidative tolerance, promoting regulated cell death pathways

Collectively, these findings demonstrate that chalcones disrupt redox homeostasis in non-small cell lung cancer through a multifaceted modulation of oxidative signaling networks. By simultaneously increasing reactive oxygen species production, impairing antioxidant defenses, and activating stress-response pathways across mitochondria and the ER, chalcones drive cancer cells beyond their oxidative tolerance threshold. Importantly, the heightened basal oxidative stress characteristic of tumor cells may underlie the preferential vulnerability of non-small cell lung cancer to chalcone-induced redox imbalance, supporting their potential as selective anticancer agents (Fig. 4).

## Chalcones modulate cell-cycle checkpoints, dna damage responses and cell death

### Cell-cycle and genotoxic damage

The cell cycle coordinates DNA replication and division through tightly regulated checkpoints that prevent genomic instability. In cancer, these checkpoints are often disrupted, leading to uncontrolled proliferation and resistance to apoptosis (Pietenpol and Stewart 2002; Suski et al. 2021; Matthews et al. 2022). Several chalcones modulate the cell cycle, frequently inducing G2/M arrest.

Natural chalcones also arrest cells at G2/M. Licochalcone A regulated more than 20 lung cancer–related genes and caused S and G2/M arrest in A549 cells (Lin et al. 2019). Panduratin A increased phospho-histone H3, confirming mitotic arrest (Cheah et al. 2011), while echinatin reduced cyclin expression in sensitive and gefitinib-resistant NSCLC cells (Oh et al. 2020a). Among nine pyrazole chalcones, PCH-1 was most active in A549 cells, causing G2/M accumulation followed by apoptosis (Maciejewska et al. 2022). Similarly, pyrazolo[1,5-a]pyrimidine chalcones (E)-1-(4-methoxyphenyl)-3-(7-(4-methoxyphenyl)-2-phenylpyrazolo[1,5-a]pyrimidin-5-yl)prop-2-en-1-one (6b), (E)-1-(3,4-dimethoxyphenyl)-3-(7-(3,4-dimethoxyphenyl)-2-phenylpyrazolo[1,5-a]pyrimidin-5-yl)prop-2-en-1-one (6 h), and (E)-3-(7-(3,4-dimethoxyphenyl)-2-phenylpyrazolo[1,5-a]pyrimidin-5-yl)-1-(3,4,5-trimethoxyphenyl)prop-2-en-1-one (6i) produced greater G2/M arrest than erlotinib and promoted cell death (Bagul et al. 2017). Butein also induced G2/M arrest in A549 and PC-9 cells by downregulating regulatory proteins (Di et al. 2019). Other derivatives, such as (E)-3-(3-Methoxy-4-(3-(piperidin-1-yl)propoxy)phenyl)-1-(4-methoxyphenyl)prop-2-en-1-one (4a) (Shankaraiah et al. 2017), indolyl-tetralone chalcone 11 (Wang et al. 2016), and oleanolic/ursolic amide analogs (Khusnutdinova et al. 2021), produced similar effects.

Some chalcones were found to induce G1 arrest. Pyrazolyl chalcone (E)-1-(3-(furan-2-yl)-5-methyl-1-(4-nitrophenyl)-1H-pyrazol-4-yl)-3-(thiophen-2-yl)prop-2-en-1-one (7b) downregulated CDK4, promoting G0/G1 accumulation (Mohamed et al. 2023), and 2′-hydroxy-2,3,4′,6′-tetramethoxychalcone increased G1 cells while reducing S-phase progression (Rao et al. 2010). Deoxycholic acid–chalcone conjugates (4b–4e) elevated sub-G1 populations, with 4b targeting CDKs, cyclins, and p53-related pathways (Patel et al. 2022). DK-139 also increased sub-G1 fractions in various NSCLC lines (Gil et al. 2019).

S-phase arrest has been observed with 2′,4,4′-trihydroxy-3′-prenyl-6′- Methoxychalcone (xanthohumol) and 2′,4,4′-trihydroxy-6′-methoxy chalcone (helichrysetin), both causing S-phase accumulation and caspase activation in A549 cells (Ho et al. 2013; Yong et al. 2015). In addition to its effects on S-phase progression, xanthohumol has been shown to suppress non-small cell lung cancer cell proliferation by targeting the ERK1/2–Fra1 signaling axis, leading to reduced cyclin D1 expression, thereby impairing cell cycle progression at earlier checkpoints (Gao et al. 2020). Cell cycle regulation involves sequential activation of cyclins and CDKs, such as cyclin D-CDK4/6 for G1, cyclin E/A-CDK2 for S-phase, and cyclin A/B-CDK1 for mitosis (Matthews et al. 2022). Chalcones modulate these checkpoints through multiple pathways: Compound 4b targets CDK2/4/6, cyclins D/E, and MDM2, influencing the p53 axis (Patel et al. 2022); Pyrazolyl chalcone 7b inhibits CDK4 (Mohamed et al. 2023); Echinatin suppresses cyclin B1 and cdc2 while inducing p27 (Oh et al. 2020a); Another indolyl-based chalcone, compound 1l, was reported to reduce cyclin A2 levels and pRb phosphorylation (Wang et al. 2016). While 2′-hydroxy-2,3,4′,6′-tetramethoxychalcone inhibits phospho-cdc2 and pRb, upregulating p53/p21 and blocking E2F-dependent transcription (Rao et al. 2010).

Microtubules, composed of α- and β-tubulin, are vital for mitosis and serve as key targets for chalcone action (Kavallaris 2010; Janke and Magiera 2020). PCH-1 disrupted tubulin polymerization, causing prolonged metaphase and multinucleation (Maciejewska et al. 2022). Compound 4a similarly inhibited tubulin assembly, with docking confirming strong α/β-tubulin interactions (Shankaraiah et al. 2017). Prenylated chalcone 2′-prenyloxy-3,4,4′,5,6′-pentamethoxy-chalcone caused monopolar spindle formation and spindle assembly checkpoint activation in NCI-H460 cells (Fonseca et al. 2016).

Chalcones also activates p53-mediated DNA damage responses (DDR). The compound 2′-hydroxy-2,3,4′,6′-tetramethoxychalcone upregulated p53 and p21, inducing G1/G2 arrest through cdc2 inhibition (Rao et al. 2010). Since p21 may act independently of p53 (Jung et al. 2014), chalcones may engage both p53-dependent and -independent mechanisms. Butein triggers DNA fragmentation and apoptosis while inhibiting inflammatory genes such as COX-2, showing broad antitumor activity (Li et al. 2014). Pyrazolyl chalcone 7 g increases DNA damage and reduces FOXG1 expression, enhancing cytotoxicity (Helmy et al. 2022). Licochalcone A suppresses Bcl-2 and PARP, while Isoliquiritigenin activates p53, Fas, and caspase cascades, restoring apoptosis in A549 cells (Hsu et al. 2004; Qiu et al. 2017; Tian et al. 2018).

**Fig. 5:**
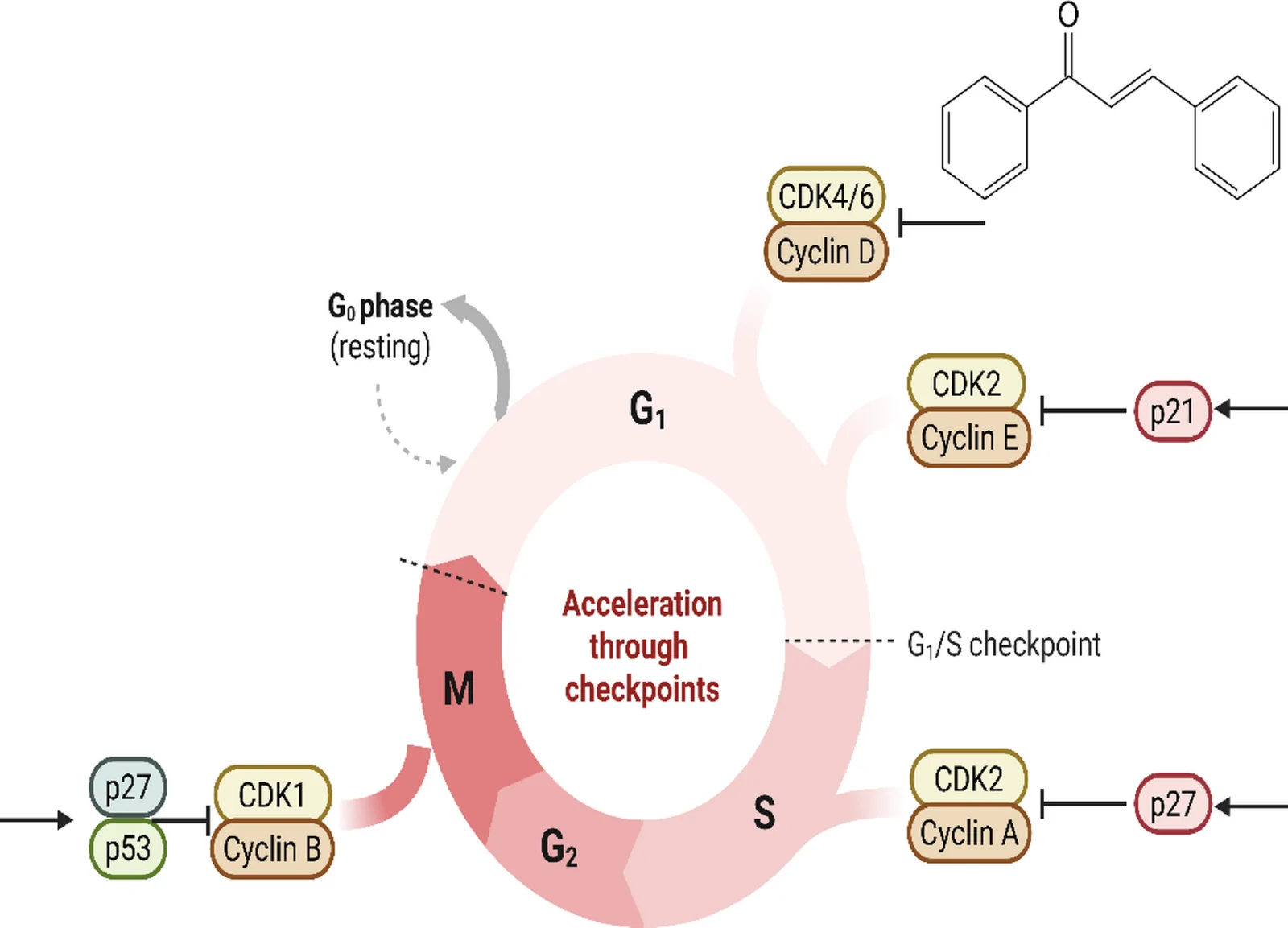
Chalcone-induced cell cycle arrest and checkpoint disruption in non-small cell lung cancer. Chalcones suppress cell proliferation in non-small cell lung cancer by disrupting key regulatory checkpoints in the cell cycle. By inhibiting cyclin–CDK complexes and upregulating cyclin-dependent kinase inhibitors such as p21 and p27, chalcones induce cell cycle arrest at the G1/S and G2/M transitions. This sustained activation of the checkpoints limits uncontrolled cell cycle progression and contributes to the anti-proliferative effects of chalcones in non-small cell lung cancer

In addition to their effects on cell cycle regulation, chalcones can directly interfere with DNA integrity and repair machinery (Leite et al. 2023). Some derivatives induce double-strand breaks or inhibit key repair enzymes such as PARP and DNA-PK, sensitizing cancer cells to genotoxic agents and radiotherapy. The generation of oxidative lesions through ROS overproduction further amplifies DNA damage, while impaired activation of homologous recombination and non-homologous end joining limits recovery of damaged cells. This dual mechanism, combining ROS-driven genotoxic stress with checkpoint disruption, forces tumor cells into mitotic catastrophe or apoptotic death. Thus, chalcones represent a unique class of multitarget compounds capable of simultaneously attacking DNA, microtubules, and cell cycle regulators, offering a strong rationale for their development as chemosensitizers or standalone agents in NSCLC therapy (Fig. 5) (Ashraf et al. 2017; Li et al. 2020; Othman et al. 2022).

### Cell death

Cell death, defined as the irreversible cessation of cellular functions, can occur as accidental cell death (ACD) or regulated cell death (RCD), also termed programmed cell death (PCD) (Majno and Joris 1995; Galluzzi et al. 2015). While ACD results from uncontrollable stress, RCD/PCD involves genetically encoded signaling pathways with distinct morphological and biochemical features (Galluzzi et al. 2018; Hänggi and Ruffell 2023; Park et al. 2023). In cancer, dysregulated RCD contributes to tumor progression and therapy resistance (Hanahan and Weinberg 2000; Galluzzi et al. 2007), making its reactivation a promising therapeutic strategy.

Several studies demonstrate the pro-death potential of chalcones in NSCLC models (Hsu et al. 2004; Mai et al. 2014; Çavuşoğlu et al. 2018; Ahmed et al. 2018; Dong et al. 2018; Lin et al. 2019; Wang et al. 2020; Chouiter et al. 2020; Qiao et al. 2020; Yang et al. 2020; Zhao et al. 2023). Licochalcone A promotes apoptosis by increasing cleaved PARP1 and caspase-3, while reducing Bcl-2 and Bcl-xL levels in A549 and H460 cells (Qiu et al. 2017). In H226 and H1703 cells, this compound triggers mitochondrial-mediated apoptosis through Bax upregulation, Bcl-2 suppression, and PARP/caspase-3 cleavage, also inhibiting ERK1/2 and p38 MAPK phosphorylation and downregulating FBXO5 (Fan et al. 2023).

Natural chalcones, such as Helichrysetin and Cardamonin, also induce apoptosis in A549 and H460 cells via DNA fragmentation and caspase-3 activation(Ho et al. 2013; Zhou et al. 2019), largely through inhibition of the PI3K/AKT/mTOR axis (Tang et al. 2014). Beyond signaling effects, chalcones interfere with microtubule dynamics. Indolyl-chalcones (CIT-026, CIT-214, CIT-223) and compound 4a inhibit tubulin polymerization and promote apoptosis (Wegiel et al. 2016; Shankaraiah et al. 2017), while imidazo[2,1-b]thiazole chalcones (11x) cause chromatin condensation and apoptotic body formation through colchicine-site binding (Kamal et al. 2014).

Apoptosis can also be triggered via death receptor signaling. 2′-Hydroxy-4′,5′-dimethoxychalcone activates DR5 and the PERK–ATF4–CHOP axis, generating ROS and suppressing c-FLIPL, which sensitizes cells to apoptosis (Yang et al. 2013). Isoxazolyl chalcones (5f–5i) further promote DR5-mediated caspase activation (Wan et al. 2014). Butein induces apoptosis through Bax and PUMA upregulation, Bcl-2 suppression, and ER stress activation via PERK/eIF2α/CHOP signaling (Di et al. 2019). Interestingly, CHOP is also linked to autophagy induction by Licochalcone A (Tang et al. 2016).

The interplay between autophagy and apoptosis is critical in tumor therapy (Maiuri et al. 2007; Gump and Thorburn 2011). Chalcones such as Flavokawain B trigger both processes by inhibiting mTOR and activating Atg-7, LC3-II, and caspases (Hseu et al. 2019). Isobavachalcone modulates the AMPK/AKT/mTOR axis, promoting autophagy and apoptosis (Wang et al. 2024), whereas Calyxin Y induces a JNK-dependent switch from autophagy to apoptosis via oxidative stress (Zhang et al. 2013).

Combining chalcones with chemotherapy enhances therapeutic outcomes. Chalcone-24 synergizes with cisplatin to trigger autophagy-associated apoptosis (Shi et al. 2015). Ferrocene–quinidine epimers increase ROS and sensitize MDR cells to paclitaxel and doxorubicin (Podolski-Renić et al. 2017). Triazinyl-chalcones combined with cisplatin also promote DNA damage and apoptosis in A549 cells (El-Wakil et al. 2020).

In the TME, chalcones modulate inflammation and immune evasion. Hydroxysafflor yellow A inhibits LPS-induced proliferation and PI3K/AKT/mTOR/ERK signaling, restoring cytokine balance in A549 and H1299 cells (Jiang et al. 2019). Panduratin A suppresses TNF-α–induced NF-κB activation and reduces MMP-2 secretion in A549 cells, thereby limiting tumor invasiveness (Cheah et al. 2011). In contrast, its structurally related derivatives, 4-hydroxypanduratin A and isopanduratin A, inhibit TNF-α–induced ICAM-1 and VCAM-1 expression by blocking NF-κB signaling in A549 cells, further contributing to the suppression of invasive phenotypes (Tanigaki et al. 2019). Butein also inhibits STAT1-mediated PD-L1 expression and enhances CD8⁺ T cell infiltration (Zhao et al. 2023).

Pyroptosis, an inflammatory PCD form, can also be triggered by chalcones. Compound 8 induces pyroptosis-like swelling and caspase-3 degradation in NCI-H460 cells (Zhu et al. 2018). Moreover, chalcones target oncogenic drivers such as EGFR, MET, KRAS, and ALK. Indolyl-tetralone chalcone 1l induces apoptosis in KRAS-mutant A549 cells (Wang et al. 2016), while Echinatin and Licochalcone D inhibit EGFR/MET signaling and activate mitochondrial apoptosis (Oh et al. 2020b). Isoliquiritigenin (ILQ) suppresses wild-type and mutant EGFR activity, reducing AKT and ERK1/2 phosphorylation in TKI-resistant NSCLC (Jung et al. 2014).

Dual EGFR/MET inhibition was also observed for butein and related derivatives, which suppress gefitinib-resistant NSCLC proliferation and promote PARP cleavage and apoptosis (Jung et al. 2015). Similarly, 3-deoxysappanchalcone was identified as a potent dual EGFR/MET inhibitor, directly interacting with both kinases as demonstrated by pull-down assays, kinase activity analyses, and molecular docking, and effectively inducing apoptosis in EGFR-mutant, MET-amplified, gefitinib-resistant NSCLC cells, including HCC827GR cells (Lee et al. 2023). In parallel, pyrazolo[1,5-a]pyrimidine chalcones (6b, 6 h, and 6i) downregulate STAT3/AKT signaling while docking efficiently into the ATP-binding site of EGFR, further supporting the potential of chalcone scaffolds as multitarget kinase inhibitors (Bagul et al. 2017).

Chalcones may also target Hsp90, destabilizing oncogenic client proteins such as EGFR, MET, and AKT. Compound 1 g suppresses proliferation and promotes degradation of these proteins while inducing Hsp70 expression in resistant NSCLC cells (Jeong et al. 2017). Moreover, Licochalcone A overcomes c-Met–mediated TKI resistance by inhibiting ERK1/2 and AKT signaling and inducing apoptosis in vitro and in xenografts (Han et al. 2022). Xanthohumol similarly reduces ERK1/2 signaling and activates caspases-3, -8, and -9 (Yong et al. 2015; Sławińska-Brych et al. 2016).

**Fig. 6:**
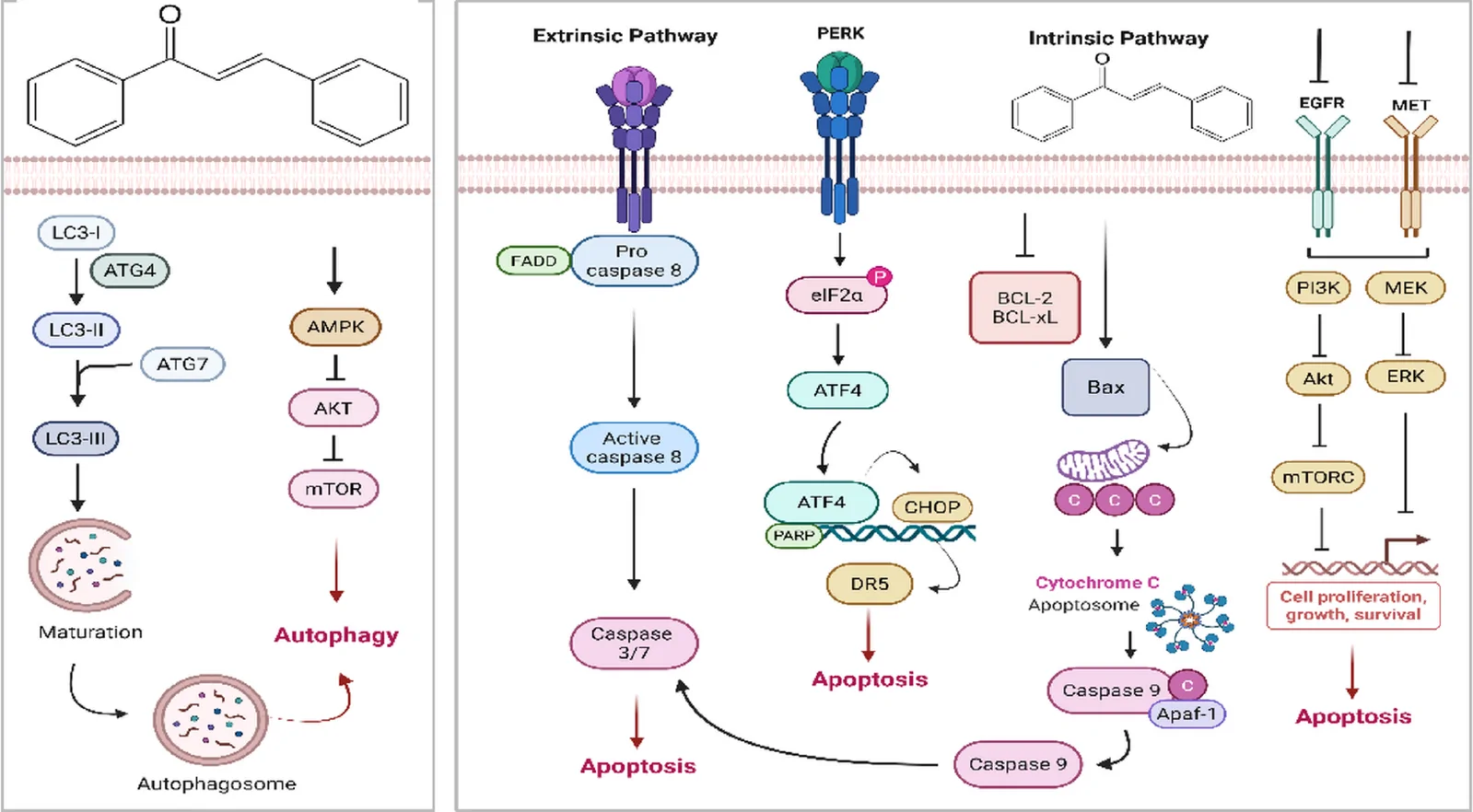
Chalcone-induced autophagy and apoptotic signaling in non-small cell lung cancer. Chalcones promote regulated cell death in non-small cell lung cancer by engaging both the autophagic and apoptotic pathways, while simultaneously suppressing pro-survival signaling networks. Chalcone-mediated activation of stress responses and inhibition of oncogenic pathways converge on death receptor signaling and mitochondrial dysfunction, leading to the activation of caspases and irreversible cell death. The coordinated disruption of survival, stress adaptation and cell fate control highlights the multitarget anticancer potential of chalcones

In summary, chalcones display multifaceted mechanisms against NSCLC, triggering apoptosis, autophagy, and pyroptosis; disrupting microtubules; inhibiting oncogenic signaling and immune evasion; and sensitizing tumors to chemotherapy. Their ability to act on multiple molecular targets and overcome resistance highlights their potential as promising scaffolds for next-generation NSCLC therapeutics (Fig. 6).

## Toxicological perspectives and selective cytotoxicity of chalcones in NSCLC

Selectivity is one of the major challenges in developing anticancer agents, since efficacy depends on eliminating tumor cells while preserving normal tissues (Schirrmacher 2018; Blagosklonny 2023). In this context, compounds with preferential cytotoxicity toward tumor cells might expand the therapeutic window and reduce the systemic toxicity frequently associated with conventional treatments (Nakhjavani et al. 2014; Hashim et al. 2024).

From the non-tumoral models, bronchial and pulmonary epithelial cells (e.g., BEAS-2B and HBE) are the most relevant systems for assessing the selectivity in NSCLC. Several studies demonstrate that chalcones exert preferential cytotoxicity in tumor cells than in these non-malignant lineages (Zhang et al. 2013; Qiu et al. 2017; Dong et al. 2018; Erturk et al. 2023; Ma et al. 2024; Di et al. 2019; Han et al. 2022; Fan et al. 2023). For instance, licochalcone A significantly reduced the viability of lung squamous carcinoma cells (H226 and H1703) while maintaining preserved viability in HBE cells (Fan et al. 2023). Furthermore, the chalcone–thiadiazole hybrid ZW97 exhibited marked selectivity, with *IC*_50_ values as low as 0.15 µM in NCI-H460 cells compared to no detectable cytotoxicity in BEAS-2B cells at concentrations exceeding 64 µM (Zhao et al. 2022).

Similar findings were observed in non-tumoral pulmonary fibroblast models (Rao et al. 2010; Zhang et al. 2013; Sławińska-Brych et al. 2016; Tang et al. 2016; Qiu et al. 2017; El-Wakil et al. 2020; Burmaoglu et al. 2021; Trávníček et al. 2023, 2024; Mohamed et al. 2023). In these systems, various chalcone exhibited reduced toxicity compared to NSCLC cell lines, suggesting that their selective function extends beyond epithelial models. Xanthohumol, for example, displayed significantly higher *IC*_50_ values in MRC-5 cells than in A549 cells (Yong et al. 2015), supporting the notion that chalcone derivatives preferentially target pathways associated with tumor survival rather than indiscriminately affecting all proliferative cell types.

This selective profile is also evident in other tissues, including hepatocytes (LO2), keratinocytes (HaCaT) and embryonic kidney cells (HEK293) (Tang et al. 2016; Chen et al. 2017; Oh et al. 2019; Helmy et al. 2022; Soumyashree et al. 2023; Kaur et al. 2019). Interestingly, some compounds showed cytoprotective role under chemically induced stress. Isoliquiritigenin, for example, reduced ROS accumulation, restored antioxidant defenses such as GSH and SOD, and activated the Nrf2 signaling pathway in hepatocytes (Wang et al. 2020). Similarly, synthetic chalcone derivatives induced detoxification enzymes, such as HO-1, without inducing cytotoxicity in normal cells (Kumar et al. 2011).

In vivo studies, using different chalcone derivatives in xenograft models, demonstrated antitumor activity no apparent systemic toxicity under the tested conditions, as evidenced by stable body weight and preserved organ morphology (Zhao et al. 2022; Liu et al. 2023b; Fan et al. 2023). Biochemical analyses showed no significant alterations in serum alanine aminotransferase (ALT), aspartate aminotransferase (AST), or blood urea nitrogen (BUN) levels, markers of hepatic and renal function (Yong et al. 2015; Zhao et al. 2022; Fan et al. 2023; Ma et al. 2024). Notably, the derivative HCTMPPK (a chlorinated chalcone), showed no detectable toxicity during prolonged administration (Ma et al. 2024). Furthermore, the panduratin A derivative DD-218 attenuated cisplatin-induced nephrotoxicity by reducing creatinine and Neutrophil Gelatinase-Associated Lipocalin (NGAL, a biomarker of kidney damage), suggesting that chalcones may actively modulate pathways associated with tissue protection (Thongnuanjan et al. 2021).

Overall, chalcone derivatives exhibit anti-tumor activity with a promising selectivity profile. Nevertheless, current data are predominantly restricted to short-term preclinical evaluations. Additional investigations addressing pharmacokinetics, biodistribution, and long-term tolerability are still required to define the translational potential of these compounds in NSCLC treatment.

## Integrated cellular responses to chalcone exposure

Although the mechanisms discussed in this review have been presented in separate sections for organizational purposes, accumulating evidence indicates that redox homeostasis, DNA damage responses (DDR), epithelial–mesenchymal plasticity, and regulated cell death constitute a highly interconnected network of adaptive processes that collectively sustain tumor survival. Rather than operating as independent pathways, these mechanisms continuously influence one another through multiple feedback loops. Therefore, understanding how chalcones affect these interconnected responses may provide a comprehensive view of their antitumor activity in NSCLC (Hanahan 2022; Cheung and Vousden 2022).

Within this framework, redox homeostasis emerges as a central point of convergence. The electrophilic α,β-unsaturated carbonyl moiety characteristic of chalcones acts as an upstream molecular trigger by reacting with intracellular thiols, including glutathione and redox-sensitive cysteine residues in regulatory proteins (Kachadourian and Day 2006; Bukhari et al. 2012; Zhuang et al. 2017). This interaction promotes ROS accumulation, which subsequently propagates through mitochondrial, genomic, and phenotypic regulatory pathways. In NSCLC, a mechanism such as this is particularly relevant, given that tumor cells already exist under chronically elevated oxidative stress due to oncogenic signaling, metabolic reprogramming, and adaptation to the TME (Cheung and Vousden 2022; An et al. 2024). Under these conditions, chalcone-induced thiol depletion does not simply increase oxidative burden; instead, it pushes an already stressed redox system beyond its adaptive capacity, transforming a survival mechanism into a source of cellular vulnerability. The consequences of this redox imbalance extend far beyond oxidative damage itself.

Mitochondria and the ER, two major hubs of cellular stress signaling, are particularly sensitive to ROS-mediated perturbations. As discussed throughout this review, chalcone treatment frequently induces mitochondrial membrane depolarization, calcium dysregulation, and additional ROS production, creating self-amplifying oxidative feedback loops. Simultaneously, ROS accumulation promotes protein misfolding and ER dysfunction, activating the unfolded protein response (UPR), particularly through the PERK/eIF2α/ATF4/CHOP axis. While these responses initially function to restore homeostasis, sustained activation progressively shifts the balance from adaptation toward irreversible cell death (Di et al. 2019; Cheung and Vousden 2022; Cao et al. 2022).

Persistent oxidative stress also has profound consequences for genomic stability. Elevated ROS levels promote oxidative DNA lesions, replication stress, and chromosomal instability, thereby activating DDR pathways (Kumar et al. 2011). In this context, the G1, S, and G2/M cell-cycle arrests frequently observed following chalcone treatment may initially represent adaptive attempts to preserve genomic integrity (Pearl et al. 2015; Hanahan 2022). However, when oxidative and genotoxic stress exceed repair capacity, checkpoint activation becomes insufficient to restore cellular homeostasis. Importantly, prolonged DDR signaling is not exclusively tumor suppressive. Under sublethal stress conditions, DDR can activate compensatory survival pathways, including NF-κB and PI3K/AKT signaling, thereby promoting stress tolerance and potentially contributing to therapy resistance (Pearl et al. 2015; Hanahan 2022). Several chalcone derivatives discussed in this review, including butein, licochalcone A, and pyrazolyl chalcone 7 g, induce DNA damage while suppressing AKT and NF-κB signaling simultaneously (Li et al. 2014; Huang et al. 2014; Qiu et al. 2017; Zheng et al. 2021; Sulaiman et al. 2021; Helmy et al. 2022). These observations suggest that part of their antitumor activity may derive from disrupting this adaptive DDR-to-survival feedback circuit, thereby increasing the likelihood of irreversible cell fate decisions.

Beyond genomic stability, oxidative stress also influences cellular phenotypic plasticity. Increasing evidence indicates that EMT should not be viewed solely as a mechanism of migration and invasion, but rather as a dynamic adaptive program that enables tumor cells to tolerate oxidative stress, therapeutic pressure, and genomic instability (Roche 2018; Bocci et al. 2019; Hwang et al. 2022). Notably, many of the signaling pathways targeted by chalcones, such as TGF-β, PI3K/AKT/mTOR, JAK/STAT, NF-κB, and Wnt/β-catenin, simultaneously regulate EMT, redox adaptation, survival signaling, and DDR. EMT is frequently associated with increased antioxidant capacity through enhanced glutathione biosynthesis, elevated Nrf2 activity, and upregulation of detoxification enzymes, collectively raising the oxidative threshold of mesenchymal-like tumor cells (Hao et al. 2019; Hu et al. 2021; Hwang et al. 2022). Consequently, the reversal of EMT by chalcones may exert effects that extend beyond inhibition of metastatic traits. By dismantling this redox-protective phenotype, chalcones reduce the oxidative tolerance of tumor cells, rendering them more susceptible to the ROS burden they generate. In this regard, ROS accumulation and EMT inhibition should not be viewed as independent phenomena but as mutually reinforcing processes that converge to amplify tumor cell vulnerability (Cheung and Vousden 2022).

Taken together, the evidence discussed throughout this review supports a model in which the antitumor activity of chalcones arises from the progressive disruption of interconnected adaptive mechanisms that sustain tumor survival. Redox imbalance functions as a central initiating event that propagates through organelle stress responses, genomic instability, epithelial–mesenchymal plasticity, and ultimately regulated cell death. Rather than representing independent biological outcomes, these responses reflect a coordinated network of stress-induced events in which disruption of one adaptive mechanism amplifies the vulnerability of others. Consequently, the multitarget activity of chalcones may arise not from the isolated modulation of multiple pathways, but from their ability to trigger a progressive collapse of the interconnected systems that collectively support tumor adaptation and survival.

## Conclusion

The evidence presented in this review indicates that chalcones and their derivatives represent chemically versatile scaffolds with relevant activity in preclinical NSCLC models. Through multiple, interconnected molecular pathways, these compounds modulate oxidative stress, epithelial–mesenchymal transition, DNA damage responses, and regulated cell death mechanisms. By modulating signaling pathways such as TGF-β, PI3K/AKT/mTOR, JAK/STAT, and Wnt/β-catenin, chalcones can inhibit tumor growth, migration, invasion, and therapy resistance. Their structural flexibility supports chemical optimization aimed at improving potency, selectivity, and pharmacokinetic behavior.

The qualitative SAR evidence discussed in this review suggests that electrophilic reactivity, aryl-ring substitution patterns, and pharmacophore hybridization are central determinants of chalcone activity in NSCLC. These same features also require careful toxicological consideration, since redox modulation, thiol reactivity, and electrophilicity may contribute to off-target effects when not properly controlled.

Current evidence remains largely restricted to in vitro studies and early-stage in vivo models. Comprehensive data on pharmacokinetics, biodistribution, long-term tolerability, genotoxicity, cardiotoxicity, hepatotoxicity, and developmental toxicity are still limited. Future studies should focus on integrating mechanistic validation, SAR-guided optimization, selective cytotoxicity assessment, and advanced delivery strategies to better define the therapeutic window of chalcone derivatives.

The available data supports the view of chalcones as promising preclinical scaffolds for NSCLC research. Their ability to affect cancer-related processes, such as oxidative stress, genomic instability, and evasion of cell death, provides a strong rationale for future investigation. Continued collaboration across medicinal chemistry, molecular oncology, pharmacology, and toxicology will be essential to determine whether chalcone-derived compounds can be optimized into safer and more selective anticancer strategies.

## Supplementary Information

Below is the link to the electronic supplementary material.

Supplementary Material 1

Supplementary Material 2

## Declarations

### Generative AI use

During the preparation of this manuscript, generative artificial intelligence tools were used to assist with language editing and improvement of clarity and organization. The authors reviewed and edited all content generated with the assistance of these tools and took full responsibility for the accuracy, originality, and integrity of the manuscript.
