# Supplementary material for: Chalcones as multitarget modulators in non-small cell lung cancer: mechanistic insights and therapeutic perspectives
Source: Arch Toxicol. 2026 Jul 14;100(11):4835–54. doi: 10.1007/s00204-026-04501-0 (PMC13694320; doi:10.1007/s00204-026-04501-0)
Supplement: Supplementary file 1 [file 204_2026_4501_MOESM1_ESM.docx]

| **Structure** | **Name/Code** | **Natural/ Synthetic** | **Cell Line(s)** | **Targets/ Mechanisms** | **Relevant Sections of Review** | **Reference** |
| --- | --- | --- | --- | --- | --- | --- |
|  | **Compound 67** | Synthetic | A549 | TGF-β1 inhibition; ↓ migration & invasion; EMT suppression | 3.1 | Jeong et al., 2019 |
|  | **CTI-82** | Synthetic | A549, HaCaT | Blocks Smad2 phosphorylation; restores E-cadherin; ↓ N-cadherin, vimentin, PAI-1, MMP-2/9; ↓ p-ERK1/2 | 3.1 | Jeong et al., 2020 |
|  | **PCH-1** 1-[3-(6-methoxy-2H-1,3-benzodioxol-5-yl)-5-(4-methoxyphenyl)-4,5-dihydro-1H-pyrazol-1-yl]ethan-1-one | Synthetic | A549 (TGF-β1-induced EMT); lung cancer cells | Inhibits TGF-β1-induced EMT (↓N-cadherin, vimentin, β-catenin, hTERT); ↓migration; G2/M arrest; ROS increase and caspase-dependent mitochondrial apoptosis; tubulin polymerization inhibitor (mitotic catastrophe) | 3.1, 3.3, 4, 5.1 | Maciejewska et al., 2022 |
|  | **ZW97** (E)-1-(4-(3-((1,3,4-thiadiazol-2-yl)thio) propoxy)phenyl)-3-(4-bromophenyl) prop-2-en-1-one | Synthetic | NCI-H460 | c-Met kinase inhibition; EMT reversal | 3.1 | Zhao et al., 2022 |
|  | **ISL/ILQ** Isoliquiritigenin | Natural  (*Glycyrrhiza* spp.;  commercially obtained) | A549; TKI-resistant NSCLC; *in vivo* models | FAK/Src inhibition; ↓ cytoskeletal remodeling & adhesion; anti-metastatic; p53/Fas/caspase activation; restored apoptosis; EGFR inhibition (WT and mutant); ↓ Akt & ERK1/2 in TKI-resistant NSCLC | 3.1, 4, 5.1, 5.2 | Hsu et al., 2004; Chen et al., 2018; Tian et al., 2018; Jung et al., 2014 |
|  | **THC** 2,4,2’,4’-Tetrahydroxychalcone | Natural  (metabolite of ISL) | A549  (*in vivo*) | FAK/Src inhibition; ↓ cytoskeletal remodeling & adhesion; anti-metastatic | 3.1 | Chen et al., 2018 |
|  | **LicA** Licochalcone A | Natural (*Glycyrrhiza inflata;* commercially obtained) | A549, NCI-H460, H226, H1703, H1299 | Sp1/Akt inhibition; ↓ MMP-1/-3 & migration/invasion;  ER stress (BiP, p-eIF2α, ATF4, CHOP); S and G2/M arrest; ↓ Bcl-2/Bcl-xL & PARP; Bax upregulation;  inhibition of ERK1/2, p38 & FBXO5;  overcomes c-Met–mediated TKI resistance | 3.1; 4, 5.1, 5.2 | Huang et al., 2014; Tang et al., 2016; Qiu et al., 2017; Lin et al., 2019; Fan et al., 2023; Han et al., 2022 |
|  | **HCTMPPK**  (E)-1-(E)-1- (2-hydroxy-5-chlorophenyl)-3- (3,5,6-trimethylpyrazin-2-yl)-2-propen-1 | Synthetic (chalcone–tetramethylpyrazine hybrid) | A549, *in vivo* tumor model | MELK inhibition; ↑ Bax & caspase-3; ↓ migration & invasion; tumor growth suppression | 3.2 | Ma et al., 2024 |
|  | **TEC** 2’,4,4’-trimethoxychalcone | Synthetic (chalcone-based drug) | BT549, A549 | JAK2/STAT3 inhibition; ↓ migration & invasion | 3.2 | Zhou et al., 2024 |
|  | **Butein** 3,4,2’,4’-tetrahydroxychalcone | Natural  (chalcone-type flavonoid; various plant sources; commercially obtained) | NSCLC cell lines (A549, PC-9, gefitinib-resistant NSCLC) | c-Src/JAK2/STAT3 inhibition; G2/M arrest; ↑ ROS & ER stress (PERK/eIF2α/CHOP); DNA fragmentation; Bax & PUMA upregulation; ↓ Bcl-2; inhibition of STAT1-mediated PD-L1; dual EGFR/MET inhibition; ↑ PARP cleavage; ↑ CD8⁺ T cell infiltration | 3.2; 4; 5.1, 5.2 | Di et al., 2019; Sulaiman et al., 2021; Zhang, Zong & Han, 2019; Jung et al., 2015; Zhao et al., 2023 |
|  | **6p** 2-methoxy-2’-hydroxy-4’-(4’’-methoxy-4’’-oxo-E-crotonyl) chalcone | Synthetic (polyoxygenated chalcone; FXR antagonist) | H460, H1975, H1299 | FXR antagonism; FXR-dependent inhibition of proliferation, migration & invasion (H460, H1975, H1299) | 3.2 | Niu et al., 2022; |
|  | **Cardamonin** 2,4-dihydroxy-6-methoxychalcone | Natural  *Alpinia katsumadai Hayata* | A549, H460 (lung cancer cells) | PI3K/Akt/mTOR inhibition; ↓ N-cadherin & ZEB1; ↑ E-cadherin; DNA fragmentation & caspase-3 activation; apoptosis | 3.2, 5.1, 5.2 | Zhou et al., 2019; Tang et al., 2014 |
|  | **HYSA** Hydroxysafflor yellow A | Natural (*Carthamus tinctorius;* chalcone-derived) | A549, H1299 (LPS-stimulated) | PI3K/Akt/mTOR & ERK/MAPK inhibition; EMT reversal; ↓ inflammatory mediators; restores cytokine balance; ↓ LPS-induced proliferation | 3.2, 4, 5.2 | Jiang et al., 2019 |
|  | **PA** Panduratin A | Natural (*Boesenbergia rotunda*) | A549 | NF-κB inhibition; ↓ MMP-2 secretion; G2/M and mitotic arrest (↑ phospho-histone H3); apoptosis | 3.3, 5.1, 5.2 | Cheah et al., 2011 |
|  | **4-hydroxypanduratin A Isopanduratin A** | Natural (rhizomes of *Boesenbergia pandurata*) | A549 | NF-κB inhibition; ↓ TNF-α–induced ICAM-1 and VCAM-1 expression; anti-inflammatory, anti-invasive | 5.2 | Tanigaki et al., 2019 |
|  | **Chalcone 9X** 4′-hydroxyl-3,4-dimethoxyl-3′-methoxylchalcone | Synthetic | Hepatic carcinoma cells; lung cancer cells | FOXM1 inhibition; ↓ migration & invasion; ↓ EMT markers | 3.3 | Dong et al., 2018; Li et al., 2022 |
|  | **7g** 1-[5-methyl-1-(4-nitrophenyl)-3-(thiophen-2-yl)-1H-pyrazol-4-yl]-  3-phenylprop-2-en-1-one | Synthetic (pyrazolyl chalcone) | Lung cancer cells | FOXG1 downregulation; Wnt/β-catenin/EMT modulation; ↑ DNA damage | 3.3, 5.1 | Zheng et al., 2021; Helmy et al., 2022 |
|    | **Complex 1**  [(4)-((1E)-3-(1)-benzofuran-2-yl)-3-oxoprop-1-en-1-yl]-2 methoxyphenyl chloroacetate  **Complex 2**  [(3)-((1E)-3-(1)-benzofuran-2-yl)-3-oxoprop-1-en-1-yl] phenyl chloroacetate | Synthetic (benzofuran-substituted chalcone derivatives) | NSCLC cell lines | Wnt/β-catenin pathway modulation; ↓ VIM/CDH2/SNAI1; ↑ E-cadherin; ↓ migration & invasion | 3.3 | Erturk et al., 2023 |
|  | **HYQ97** Tert-butyl-4-((2-(1-(4-cinnamoylphenyl)-1H-1,2,3-triazol-4-yl)ethanethioyl)thio)piperazine-1-carboxylate | Synthetic  (triazole–dithiocarbamate hybrid) | A549 | Hsp90 inhibition; ↓ N-cadherin & vimentin; ↑ E-cadherin; ↓ migration & invasion | 3.3 | Hu et al., 2021 |
|  | **IBC** Isobavachalcone | Natural  (*Psoralea corylifolia* L ; commercially obtained) | Breast cancer cells; lung cancer cells; *in vivo* tumor model | ROS overproduction; mitochondrial Ca²⁺ overload & depolarization; necrotic death; AMPK/AKT/mTOR modulation; autophagy and apoptosis | 4, 5.2 | Xiao et al., 2023; Wang et al., 2024 |
|  | **3a** 3-[4-(dimethylamino)phenyl]-1-(7-ethoxy-1-benzofuran-2-yl)prop-2-en-1-one | Synthetic | Cancer cell lines | ROS-dependent mitochondrial dysfunction; apoptosis | 4 | Coskun et al., 2017 |
|  | **DK-139** 2-hydroxy-3′,5,5′-trimethoxychalcone | Synthetic | Cancer cell lines | ROS accumulation; apoptosis; ↑ sub-G1 population | 4, 5.1 | Gil et al., 2019 |
|  | **TSHDC** 4-(p-toluenesulfonylamino)-3,4-dihydroxychalcone | Synthetic | Cancer cell lines | ROS accumulation via c-Myc activation; oxidative stress-dependent cytotoxicity | 4 | Kim et al., 2010 |
|  | **FKB** Flavokawain B 2′-Hydroxy-4′,6′-dimethoxychalkone | Natural  (rhizomes of *Alpinia pricei* Hayata) | A549, H1299 | ↑ROS; mTOR inhibition; LC3-II accumulation; ATG4B modulation; autophagy-associated apoptosis | 4, 5.2 | Hseu et al., 2019 |
|  | **17a** **(**OC-6-33)-Diamminetrichlorido((E)-3-(4-(3-oxo-3-(3,4,5  trimethoxyphenyl)prop-1-en-1-yl)-1H-indol-1-yl)  propanoate)platinum(IV) | Synthetic (platinum–chalcone hybrid) | A549/CDDP (cisplatin-resistant) | ROS accumulation; mitochondrial depolarization; ER stress | 4 | Liu et al., 2023 |
|  | **HDMC** 2'-hydroxy-4',5'-dimethoxychalcone | Natural  (isolated from *Sarcandra hainanensis*) | H157, H460, H1792, H322M, H358 | ↑ ROS; ER stress (PERK/p-eIF2α/ATF4/CHOP and IRE1α/XBP1); ↑ DR5; caspase-8/-9/-3 activation; ↓ c-FLIP; apoptosis and G2/M arrest; sensitization to TRAIL | 4, 5.2 | Yang et al., 2013 |
|  | **9d** 1-(1′-(4-Bromophenyl)-3′-(4-methoxyphenyl)-5-(thiophen-2-yl)-3,4-dihydro-1′H,2H-[3,4′-bipyrazol]-2-yl)ethan-1-one | Synthetic (pyrazoline chalcone) | A549 | Cytotoxicity in A549; DPPH radical scavenging (antioxidant activity) | 4.1 | Rana et al., 2023 |
|  | **2b** (E)-1-(2-methoxyphenyl)-3-(2-(trifluoromethyl)phenyl) prop-2-en-1-one | Synthetic | BEAS-2B; *in vivo* | Nrf2 activation; ↑ HO-1, NQO1, GCLM (antioxidant response) | 4.1 | Kumar et al., 2011 |
|  | **3d** 1, 1'-(3-methyl-1H-indole-1, 2-diyl) bis (3-phenylprop-2-en-1-one) | Synthetic | A549 | ↑ ROS; Nrf2/HO-1 activation; apoptosis induction | 4.1 | Zhao et al., 2017 |
|  | **2′-HC** 2′-Hydroxychalcone | Synthetic (hydroxychalcone) | A549 | Up to 90% GSH depletion; ↑ mitochondrial ROS; sensitizes to oxidative stress & cisplatin | 4.1 | Kachadourian & Day, 2006; Kachadourian, Leitner & Day, 2007 |
|  | **2′,5′-DHC** 2′,5′-Dihydroxychalcone | Synthetic (hydroxychalcone derivative) | A549 | GSH depletion; ↑ mitochondrial ROS; potentiates cisplatin cytotoxicity | 4.1 | Kachadourian, Leitner & Day, 2007 |
|  | **4′-HC** 4′-Hydroxychalcone | Synthetic (hydroxychalcone derivative) | A549 | GSH depletion; ↑ mitochondrial ROS; potentiates cisplatin cytotoxicity | 4.1 | Kachadourian, Leitner & Day, 2007 |
|  | **6b**  (E)-1-(4-Methoxyphenyl)-3-(7-(4-methoxyphenyl)-2-phenylpyrazolo[1,5-a]pyrimidin-5-yl)prop-2-en-1-one  **6h**  (E)-1-(3,4-Dimethoxyphenyl)-3-(7-(3,4-dimethoxyphenyl)-2-phenylpyrazolo[1,5-a]pyrimidin-5-yl)prop-2-en-1-one  **6i**  (E)-3-(7-(3,4-Dimethoxyphenyl)-2-phenylpyrazolo[1,5-a]pyrimidin-5-yl)-1-(3,4,5-trimethoxyphenyl)prop-2-en-1-one | Synthetic (pyrazolo[1,5-a]pyrimidine chalcones) | A549 and other NSCLC cells | Strong G2/M arrest (greater than erlotinib); cell death induction; STAT3/Akt downregulation; efficient docking at EGFR ATP-binding site | 5.1, 5.2 | Bagul et al., 2017 |
|  | **4a** (E)-3-(3-Methoxy-4-(3-(piperidin-1-yl)propoxy)phenyl)-1-(4-methoxyphenyl)prop-2-en-1-one | Synthetic | A549 (and other cancer cells) | G2/M arrest; tubulin polymerization inhibition; α/β-tubulin binding (docking) | 5.1 | Shankaraiah et al., 2017 |
|  | **1L**  indolyl-tetralone chalcone | Synthetic (indolyl-tetralone chalcone) | A549 | G2/M arrest; ↓ cyclin A2; ↓ pRb phosphorylation; apoptosis. | 5.1, 5.2 | Wang et al., 2016 |
|  | **13**  N-(4-Aminophenyl)-4-(3-(3,4-dihydroisoquinolin-2(1H)-yl)prop-1-en-2-yl)benzamide | Synthetic (oleanolic/ursolic amide–chalcone analogs) | Lung cancer cells | G2/M arrest; cell cycle disruption | 5.1 | Khusnutdinova et al., 2021 |
|  | **ECN** Echinatin | Natural (*Glycyrrhiza* spp.) | NSCLC cells, including gefitinib-resistant | G2/M arrest; ↓ cyclin B1 & cdc2; ↑ p27 | 5.1 | Oh et al., 2020a |
|  | **7b**  (E)-1-(3-(Furan-2-yl)-5-methyl-1-(4-nitrophenyl)-1H-pyrazol-4-yl)-3-(thiophen-2-yl)prop-2-en-1-one | Synthetic (pyrazolyl chalcone) | Lung cancer cells | CDK4 inhibition; G0/G1 arrest; ↓ cyclin D/CDK4 signaling | 5.1 | Mohamed et al., 2023 |
|  | **HTMC** 2′-hydroxy-2,3,4′,6′-tetramethoxychalcone | Synthetic (chalcone derivative) | Lung cancer cells | G1 arrest; ↓ phospho-cdc2 & pRb; ↑ p53/p21; E2F-dependent transcription blockade | 5.1 | Rao et al., 2010 |
|  | **4b–4e** Deoxycholic acid–chalcone conjugates | Synthetic (deoxycholic acid–chalcone conjugates) | Lung cancer cells | ↑ sub-G1 population; 4b inhibits CDK2/4/6, cyclins D/E & MDM2; p53 axis modulation | 5.1 | Patel et al., 2022 |
|  | **Xanthohumol** 2’,4,4’‑trihydroxy‑3’‑prenyl-6’‑methoxychalcone | Natural  (*Humulus lupulus*) | A549; HCC827; H1975; H23 | Cell-cycle arrest (S phase, G0/G1); ↓ ERK1/2–Fra1–cyclin D1 axis; caspase-3/-8/-9-mediated apoptosis; ↓ ERK1/2 signaling. | 5.1, 5.2 | Yong et al., 2015; Sławińska-Brych et al., 2016; Gao et al., 2020 |
|  | **Helichrysetin** 2′,4,4′-trihydroxy-6′-methoxy chalcone | Natural (Helichrysum-derived chalcone) | A549, H460 | S-phase arrest; DNA fragmentation; caspase-3 activation; PI3K/Akt/mTOR inhibition | 5.1, 5.2 | Ho et al., 2013; Yong, Ho & Malek, 2015; Tang et al., 2014 |
|  | **PC2** 2′-prenyloxy-3,4,4′,5,6′-pentamethoxy-chalcone | Synthetic (prenylated chalcone) | NCI-H460 | Monopolar spindle formation; spindle assembly checkpoint activation; mitotic arrest | 5.1 | Fonseca et al., 2016 |
|      | **CIT-214**  (2E)-7-fluoro-2-(1H-indol-5-ylmethylidene)-1,2,3,4-tetrahydronaphthalen-1-one  **CIT-026**  (2E)-7-fluoro-2-[(1-methyl-1H-indol-5-yl)methylidene]-1,2,3,4-tetrahydronaphthalen-1-one  **CIT-223**  (2E)-2-[(1-ethyl-1H-indol-5-yl)methylidene]-7-fluoro-1,2,3,4-tetrahydronaphthalen-1-one | Synthetic (indolyl chalcones) | NSCLC cell lines | Tubulin polymerization inhibition; microtubule disruption; apoptosis | 5.2 | Wegiel et al., 2016 |
|  | **11X**  (E)-3-(6-(4-fluorophenyl)-2,3-bis(4-methoxyphenyl)imidazo[2,1-b]thiazol-5-yl)-1-(pyridin-2-yl)prop-2-en-1-one | Synthetic (imidazo-thiazole chalcones) | NSCLC cell lines | Colchicine-site binding; chromatin condensation; apoptotic body formation | 5.2 | Kamal et al., 2014 |
|    | **5f**  3-(2-Chlorophenyl)-1-(5-methyl-3-(4-nitrophenyl)isoxazol-4-yl)prop-2-en-1-one**,**  **5g**  3-(4-Chlorophenyl)-1-(5-methyl-3-(4-nitrophenyl)isoxazol-4-yl)prop-2-en-1-one  **5h**  3-(2-Bromophenyl)-1-(5-methyl-3-(4-nitrophenyl)isoxazol-4-yl)prop-2-en-1-one  **5i**  3-(4-Bromophenyl)-1-(5-methyl-3-(4-nitrophenyl)isoxazol-4-yl)prop-2-en-1-one | Synthetic (isoxazolyl chalcones) | Lung cancer cells | DR5-mediated caspase activation; apoptosis | 5.2 | Wan et al., 2014 |
|  | **Calyxin Y** | Natural (chalcone derivative) | Lung cancer cells | Oxidative stress; JNK-dependent switch from autophagy to apoptosis | 5.2 | Zhang et al., 2013 |
|  | **Chal-24** | Synthetic | Lung cancer cells (with cisplatin) | Synergy with cisplatin; autophagy-associated apoptosis | 5.2 | Shi et al., 2015 |
|   **4**    **5**    **6**    **7** | **4,5,6 and 7** Ferrocene–quinidine epimers | Synthetic (ferrocene–quinidine hybrids) | MDR cancer cells (including NSCLC subtypes) | ↑ ROS; sensitization to paclitaxel and doxorubicin; apoptosis | 5.2 | Podolski-Renić et al., 2017 |
|    | **10**  (E)-2,2′-{[6-(4-(3-(4-Clorophenyl)prop-2-enoyl)anilino)-1,3,5-triazine-2,4-diyl]diazanediyl} bis(3-methylbutanoic acid)  **12**  (E)-2,2′-{[6-(4-(3-(4-Clorophenyl)prop-2-enoyl)anilino)-1,3,5-triazine-2,4-diyl]diazanediyl} bis(3-phenylpropanoic acid) | Synthetic (triazinyl–chalcone hybrids) | A549  (± cisplatin) | Combined with cisplatin; ↑ DNA damage; apoptosis in A549 | 5.2 | El-Wakil et al., 2020 |
|  | **8** (E)-N-(4-(3-(2-chlorophenyl)acryloyl)phenyl) acrylamide | Synthetic | NCI-H460, A549, H1975 | ROS overproduction; caspase-3–dependent pyroptosis; ↓ pro-caspase-3; growth inhibition | 4, 5.2 | Zhu et al., 2018 |
|  | **LicD**  (E)-3-(3,4-dihydroxy-2-methoxyphenyl)-1-[4-hydroxy-3-(3-methylbut-2-enyl)phenyl]prop-2-en-1-one | Natural (*Glycyrrhiza* spp.) | EGFR/MET-driven NSCLC cells | EGFR/MET inhibition; mitochondrial apoptosis | 5.2 | Oh et al., 2020b |
|  | **(3-DSC)**  3-Deoxysappanchalcone | Natural (*Caesalpinia sappan*; synthesized for this study) | HCC827GR (gefitinib-resistant NSCLC) | Dual EGFR/MET inhibition (↓p-EGFR/p-MET, ↓AKT/ERK); G2/M & sub-G1 arrest; ROS- and ER-stress–mediated, mitochondria-dependent caspase apoptosis | 5.2 | Lee et al., 2023 |
|  | **1g** | Synthetic | Resistant NSCLC cells | Hsp90 inhibition; EGFR/MET/Akt degradation; ↑ Hsp70; ↓ proliferation | 5.2 | Jeong et al., 2017 |
