# Supplementary material for: Chalcones as multitarget modulators in non-small cell lung cancer: mechanistic insights and therapeutic perspectives
Source: Arch Toxicol. 2026 Jul 14;100(11):4835–54. doi: 10.1007/s00204-026-04501-0 (PMC13694320; doi:10.1007/s00204-026-04501-0)
Supplement: Supplementary file 2 [file 204_2026_4501_MOESM2_ESM.docx]

| **Structural determinant** | **Representative compounds** | **Primary mechanistic tendency in NSCLC** | **Functional role in SAR hierarchy** | **Structure–activity  interpretation** | **Toxicological implication** |
| --- | --- | --- | --- | --- | --- |
| α,β-unsaturated carbonyl  (Michael acceptor) | 2′-HC, 2′,5′-DHC, 4′-HC, chalcone core | GSH depletion; ROS accumulation; apoptosis; sensitization to chemotherapy | Core reactivity driver | Central electrophilic motif enabling covalent thiol targeting and redox modulation; initiates ROS-dependent signaling cascades | Excess electrophilicity may promote nonspecific thiol reactivity, off-target protein modification, and systemic cytotoxicity |
| Polyhydroxylation (OH groups) | Butein, THC, cardamonin, helichrysetin | ROS modulation; ER stress activation; apoptosis; cell-cycle arrest | Redox modulator | Hydroxyl groups enhance hydrogen bonding and redox cycling capacity, facilitating ROS-mediated signaling and stress responses | May exhibit dual antioxidant/pro-oxidant behavior depending on dose and cellular context; potential for redox imbalance in normal cells |
| Methoxylation (OCH₃ groups) | HTMC, Flavokawain B, DK-139, 6b/6h/6i | Cell-cycle arrest; modulation of mTOR/STAT3/Akt pathways; autophagy and apoptosis | Pharmacokinetic and target-interaction modulator | Methoxy groups increase lipophilicity and membrane permeability, potentially improving target engagement and intracellular accumulation | Increased lipophilicity may alter pharmacokinetics, metabolic stability, and off-target tissue exposure |
| Halogenation  (Cl, Br, F) | ZW97, compound 8, 9d, 5f–5i | Kinase inhibition (e.g., c-Met); ROS-mediated cell death; caspase activation | Electronic and binding modulator | Halogens influence electronic distribution, molecular conformation, and binding orientation, modulating reactivity and target interaction | Effects are context-dependent; may affect selectivity and metabolic stability, requiring compound-specific toxicological evaluation |
| Nitroaromatic substitution  (NO₂ groups) | 7b, 7g, nitro-isoxazolyl chalcones | Cell-cycle arrest; DNA damage-related signaling; CDK4 modulation; DR5 activation | Reactivity enhancer / structural alert | The strong electron-withdrawing character of nitro groups can alter electronic distribution, electrophilic behavior, and redox-related responses, potentially influencing ROS generation and DNA damage-associated signaling. | Nitroaromatic moieties represent structural alerts for genotoxicity; metabolic activation may generate reactive intermediates; dedicated genotoxicity assays are required |
| Pharmacophore hybridization | PCH-1, HYQ97, HCTMPPK, 17a, 3-DSC | EMT suppression; Hsp90 inhibition; tubulin disruption; kinase modulation; ROS/ER stress induction | Targeting enhancer | Hybridization expands molecular recognition and enables multi-target engagement beyond the chalcone core | Additional pharmacophores may introduce new toxicity profiles; safety cannot be inferred solely from the chalcone scaffold |
